# Stakeholder perspectives on HPV vaccination uptake among Aboriginal and Torres Strait Islander adolescents via the school immunisation programmes in Queensland: a qualitative study

**DOI:** 10.1136/bmjopen-2024-097518

**Published:** 2025-06-04

**Authors:** Ami Morseu-Diop, Tamara Butler, Kate Anderson, Julia Brotherton, Joan Cunningham, Allison Jaure, Gail Garvey, Evan AhWing, Vanessa Clements, Sonya Egert, Frances Lomas, Casey Ross, Lisa J Whop

**Affiliations:** 1 Yardhura Walani, National Centre for Aboriginal and Torres Strait Islander Wellbeing Research, Australian National University, Canberra, Australian Capital Territory, Australia; 2 The School of Public Health, Faculty of Medicine, The University of Queensland, Brisbane, Queensland, Australia; 3 Menzies School of Health Research, Charles Darwin University, Darwin, Northern Territory, Australia; 4 Melbourne School of Population and Global Health, University of Melbourne VCCC, Melbourne, Victoria, Australia; 5 Sydney School of Public Health, The University of Sydney, Sydney, New South Wales, Australia; 6 Northwest Indigenous Catholic Social Services AU, Mt Isa, Queensland, Australia; 7 Queensland Health, Brisbane, Queensland, Australia; 8 Southern Queensland Centre of Excellence in Aboriginal and Torres Strait Islander Primary Health, Brisbane, Queensland, Australia; 9 Inala Wangarra, Brisbane, Queensland, Australia; 10 Education Queensland, Brisbane, Queensland, Australia

**Keywords:** Australian Aboriginal and Torres Strait Islander Peoples, HPV Vaccine, Immunization Programs, Health Equity, QUALITATIVE RESEARCH, Adolescents

## Abstract

**Abstract:**

**Introduction:**

Aboriginal and Torres Strait Islander women experience inequitable cervical cancer outcomes including higher incidence and mortality rates than other Australian women. Cervical cancer can be prevented through human papillomavirus (HPV) vaccination, which is primarily delivered through school immunisation programmes and found to be very effective. However, Aboriginal and Torres Strait Islander adolescents have lower rates of HPV vaccination uptake compared with non-Indigenous adolescents.

**Objectives:**

This study explored the perspectives and experiences of HPV vaccination programme providers and school staff involved in the delivery of school-based HPV vaccination programmes for Aboriginal and Torres Strait Islander adolescents in Queensland.

**Design:**

This qualitative project recruited 10 maximally diverse schools to participate. We purposively invited immunisation programme providers and school staff associated with delivering or supporting and used a snowballing approach to recruitment. We used an Indigenist Research approach and an ecological model for health to centre Aboriginal and Torres Strait Islander experiences and priorities.

**Participants:**

We interviewed 18 immunisation programme providers and school staff involved in delivery between 2020 and 2022. Interview topics included programme delivery and processes, engagement with Aboriginal and Torres Strait Islander adolescents and caregivers, factors impacting uptake and completion, and suggestions for improvement.

**Results:**

Stakeholders highlighted multilayered challenges navigating a school-based immunisation programme across health and education sectors, especially within the context of the COVID-19 pandemic. This included logistical barriers around programme coordination and scheduling, roles and responsibilities, and communication issues between schools, programme providers, caregivers and adolescents. Four themes were identified: (1) co-ordination of the clinic between schools and programme providers, (2) supporting Aboriginal and Torres Strait Islander families through the vaccination pathway, (3) HPV vaccination resources and (4) COVID-19 disruptions to HPV vaccination programme.

**Conclusions:**

The findings suggest a need for better communication and coordination of the school-based clinic, including consideration of staff capacity and school resources; enhanced linkages with and support for Aboriginal and Torres Strait Islander student support staff and community organisations who play a critical role in supporting adolescents’ vaccination, and flexible methods of consent supported by culturally appropriate resources. These findings informed recommendations for improved practice and will contribute towards reaching Australia’s cervical cancer elimination targets.

**STRENGTHS AND LIMITATIONS OF THIS STUDY**

- Research led by Aboriginal and Torres Strait Islander researchers and guided by an Aboriginal and Torres Strait Islander Steering Committee.
- Study is limited to the Queensland school immunisation programme; additional research is required to capture perspectives from key stakeholders in other States and Territories.
- Data collection occurred during the COVID-19 pandemic, as such, some perspectives from participants do not represent a ‘normal day’.

## Background

 Cervical cancer is highly preventable through the human papillomavirus (HPV) vaccine. Australia is currently leading in vaccination rates globally and is expected to become the first country to eliminate cervical cancer as a public health problem.^1^,^3^ However, Aboriginal and Torres Strait Islander people are at risk of being ‘left behind’ as they continue to experience disproportionately high rates of cervical cancer compared with other Australians (12.3 vs 5.6 per 100 000 women, respectively).^4^ HPV vaccination has been available since 2007 through Australia’s National Immunisation Program (NIP), which can be accessed for free via school-based immunisation programmes (SIP), which are coordinated by state and territory health departments. It was initially offered to female adolescents aged 12–13 years old, with the inclusion of adolescent males from 2013.

The programme moved from a three-dose quadrivalent Gardasil vaccine to a two-dose nonavalent vaccine in 2018.^4^ From February 2023, the NIP moved from a two-dose schedule for adolescents aged 12–13 years old, to a one-dose schedule utilising the same Gardasil 9 vaccine. This change in vaccine schedule was recommended by the WHO Strategic Advisory Group of Experts on Immunisation in 2022 and endorsed by the Australian Technical Advisory Group on Immunisation (ATAGI) in line with international scientific evidence on the efficacy of one dose.^5 6^ Vaccination initiation rates for Aboriginal and Torres Strait Islander adolescent males and females peaked in 2020 (83% vs 87%), however, since 2021 this has continued to decline.^2 7^

Indigenous adolescents receiving at least one dose of HPV vaccine before their 15th birthday included 80.9% of girls and 75.0% of boys in 2023, which was down from 83.0% to 78.1%, respectively, in 2022.^8^ In contrast, in 2023, all adolescents (both Indigenous and non-Indigenous) 84.2% of girls and 81.8% of boys had received at least one dose of HPV vaccine before their 15th birthday, down from 85.3% to 83.1%, respectively, in 2022.^7^ The updated one-dose schedule hoped to increase equitable HPV vaccination coverage; however, it is essential that opportunities to catch-up adolescents who miss the HPV vaccination are not missed.

Achieving equitable HPV vaccination is vital in both national and international efforts to eliminate cervical cancer. In 2020, the WHO announced a call to action to accelerate the global elimination of cervical cancer as a public health problem by 2030.^9^ As part of the three elimination pillars, there is a target to achieve HPV vaccination among 90% of girls by the age of 15 years. Here, in Australia, the National Strategy for the Elimination of Cervical Cancer^10^ sets a target for 90% of both boys and girls vaccinated against HPV. The National Strategy also outlines strategic priorities for Aboriginal and Torres Strait Islander populations. These include improving the delivery of school-based HPV immunisation programmes in all states and territories to achieve equity and high coverage.^10^ It highlights the need to review and revise HPV resources, to use alternative methods of communication through technology for reminders and follow-up, and to maximise coverage and catch-up opportunities for implementation of the one-dose schedule. Similarly, the National Immunisation Strategy for Australia 2019–2024^11^ and current developments towards the National Immunisation Strategy 2025–2030^12^ outline key priorities for improving vaccine coverage across Australia, particularly for vaccine preventable diseases. A core part of the strategy focuses on actions to improve coverage and uptake among adolescents via school-based vaccination programmes and highlights Aboriginal and Torres Strait Islander adolescents as a priority group for vaccine coverage, addressing it as an important contribution to reducing the health disparities in Indigenous health outcomes. To achieve these recommendations, it is critical that all key programme providers are consulted to understand the barriers and enablers to reaching 90% HPV vaccination coverage for all population groups in Australia.

Some of the roles of immunisation providers involved in delivering the SIP include coordinating and planning vaccination schedules with schools, educating and communicating with parents, students and school staff about the vaccines and addressing concerns, and managing adverse events on vaccination day. Postvaccination includes updating student records on the Australian Immunisation Register and organising catch-up clinics for students who missed the scheduled vaccination dates. School staff involved in delivering the SIP share similar duties, particularly around providing information to parents and students, distributing and obtaining consent forms, managing students’ emotions on vaccination day and following up with absent students.

There are limited data that explore stakeholder perspectives on HPV vaccine uptake among Aboriginal and Torres Strait Islander adolescents within the SIP. Swift *et al*^13^ examined stakeholder perspectives on the Australian HPV vaccination programme, broadly with some findings relating to Aboriginal and Torres Strait Islander adolescents. Stakeholders’ strategies for achieving high HPV vaccination coverage among Aboriginal and Torres Strait Islander students included engaging school staff, utilising Aboriginal Liaison Officers, and partnering with community organisations and Aboriginal Medical Services (AMSs). Direct communication with adolescents and families, support from community Elders and incentives like T-shirts were also perceived to be beneficial. Addressing language barriers with interpreters and providing culturally appropriate information and consent materials were crucial. Simplifying consent forms helped facilitate parental consent. Challenges included high absenteeism, mobile population and misconceptions about the vaccine. Planned initiatives specifically for Aboriginal and Torres Strait Islander adolescents included dedicated nurse immunisers, extended catch-up vaccination clinics.

A study^14^ in Aotearoa New Zealand surveyed school staff from 14 diverse schools to understand their views towards the upcoming implementation of the school-based HPV vaccination programme. The findings highlighted that school staff believed improving information and consent forms would increase vaccine uptake for Indigenous and Pacific communities, who have the highest rates of cervical cancer in New Zealand. Educating parents about the vaccine, engaging with community groups and expanding vaccine availability to community settings, schools and primary care were also highly rated.

A systematic review^3^ of 44 studies on organisational factors affecting school-based vaccination programmes in the UK and USA found workforce capacity to be a key barrier to successful programme delivery. Staff involved in delivery reported having limited time or resources to gain consent and manage the concerns of adolescents and caregivers. Delivery staff were frequently overburdened by the increased workload created by vaccination programmes. Strong initiative and efforts from both school and delivery staff were seen to be the main drivers of high return of consent forms and increased uptake. Evidence demonstrating the crucial role of school and delivery staff in SIPs also highlights the importance of understanding the views of Aboriginal and Torres Strait Islander staff involved in similar efforts.

A focused exploration of stakeholders’ perspectives on the delivery and accessibility of HPV vaccination for Aboriginal and Torres Strait Islander adolescents is needed to address programmatic barriers to HPV vaccination for this population. Understanding these key stakeholders’ perspectives is one of many key voices required to design and implement changes to the programme to better reach Aboriginal and Torres Strait Islander adolescents. An effective SIP could significantly lower the high incidence and mortality rates of cervical cancer among Aboriginal and Torres Strait Islander populations.^15^ Therefore, it is necessary to identify the specific barriers and facilitators to vaccination delivery, to achieve elimination equitably. The Yarning about HPV vaccination study^16^ was conducted in 10 schools in the state of Queensland, Australia and aimed to provide a comprehensive understanding of factors influencing HPV vaccination uptake and completion among Aboriginal and Torres Strait Islander adolescents. The aim of this study is to explore the perspectives and experiences of HPV vaccination programme providers and school staff involved in the delivery of school-based HPV vaccination programmes for Aboriginal and Torres Strait Islander adolescents in Queensland. It is one component of a larger study,^13^ which recruited 65 Aboriginal and Torres Strait Islander adolescents and 20 caregivers across 10 schools, to understand their experiences engaging with the HPV vaccination programme. We encourage readers to engage with this paper in conjunction with the broader study, for a comprehensive view.

## Methods

### Study approach

As described in the study protocol,^16^ this study was guided by an Indigenist Research approach, defined as ‘research by Indigenous people whose primary informants are Indigenous people and whose goals are to serve and inform the Indigenous struggle for self-determination’.^17^ The project was also guided by an ecological model for health promotion,^18^ which views health behaviour as a combination of factors that relate to intrapersonal, interpersonal, institutional, community and public policy.

Reporting follows the Consolidated criteria for reporting qualitative research (see online supplemental file 1)^19^ including the Consolidated criteria for strengthening reporting of health research involving Indigenous peoples (see online supplemental file 2).^20^

### Governance

As described elsewhere,^16^ a steering committee made up of Aboriginal and Torres Strait Islander people with expertise and experience in Aboriginal and Torres Strait Islander health and research was formed for the duration of the project. The steering committee provided feedback and guidance across all stages of the project. This ensured that the project was culturally safe and appropriate to meet the needs and values of Aboriginal and Torres Strait Islander people and communities. Steering committee members were remunerated for their time and invited for coauthorship.

### Reflexivity

The research team acknowledges the significant role of identity and reflexivity in conducting qualitative research and research with Aboriginal and Torres Strait Islander peoples. The multidisciplinary team consists of Aboriginal and Torres Strait Islander researchers (AM-D, TB, GG, LJW and the steering committee—EW, VC, SE, FL, and CR) and non-Indigenous (KA, JB, JC, and AJ) people with expertise in cervical cancer elimination (AM-D, TB, LJW, JB, GG, JC, AJ), community engagement (LJW, GG, steering committee) and qualitative research (AM-D, TB, KA, LJW, AJ, GG). Further details providing the research team and the steering committee reflexivity statements are reported elsewhere,^21^ collectively sharing a passion for improving health among Aboriginal and Torres Strait Islander peoples. The steering committee was a crucial element of our research governance and provided valuable knowledge and guidance informed by their professional backgrounds and lived experience as Aboriginal and/or Torres Strait Islander peoples.

### Recruitment and participants

We use ‘providers’ to refer to individuals involved in delivering and coordinating school immunisation programmes, such as nurses and immunisers, programme coordinators, council coordinators and state health department representatives. We use ‘school staff’ to refer to individuals working within the education system who are involved in supporting and facilitating the school immunisation programme, including teachers, school nurses, administration staff, school principals and student support staff. We use key informants to refer to both providers and school staff collectively.

Provider and school staff recruitment was purposive and used a snowballing approach, following consent provided by school principals for schools to participate in the study. As described elsewhere,^16^ school recruitment aimed to achieve a maximally diverse sample across Queensland (including metropolitan, regional, remote districts, government, independent schools and varying school size). Potential participants were initially identified by the nominated school representative at each participating school. The school representative provided advice on local protocols and appropriate research approach, or this was sought from other Aboriginal and Torres Strait Islander staff members. The research team approached key informants and informed them about the study that was taking place at the school. The school representative then passed on their contact details to the research team if they expressed interest in participating. Because participants chose to take part by contacting the research team, we were unable to record how people declined to participate. The research team provided all formal study information and official invitations to prospective participants via email and/or phone. Participants were also identified via the SIP coordinators, Queensland education contacts, research team and steering committee contacts, through a search of state government websites. Recruitment continued until all identified potential participants had been invited to participate. No participant dropped out or withdrew statements throughout the duration or completion of the project. As we aimed to recruit a maximally diverse sample, data saturation was not a priority. However, when analysis commenced it was clear data saturation had been achieved.

### Data collection

Research occurred between October 2020 and May 2022. While Yarning methods were used for other participant groups in the larger project, few key informants in this substudy identified as Aboriginal and Torres Strait Islander people and so semistructured interviews were used. Whenever possible, interviews were conducted privately in-person and scheduled to coincide with the SIP clinic. Interviews were also conducted over the phone or online via Zoom. All interviews were audio-recorded. Field notes were also collected during observation of the school’s immunisation clinic to provide context for the interviews (reported elsewhere^21^).

Participants provided either physical signed consent, audio-recorded verbal consent or consent via email and completed a brief demographic survey that included questions about their age, sex/gender, Indigenous status, current role, length of time in role, work experience, level of education and qualifications attained.

The researchers introduced themselves and the research topic before commencing the interview. An interview topic guide (online supplemental file 3) was used for semistructured interviews; the topics included the programme processes such as consent, programme engagement with Aboriginal and Torres Strait Islander adolescents and caregivers, information, education and promotion of the HPV vaccine, and factors that impact uptake and completion of the HPV vaccine course among Aboriginal and Torres Strait Islander adolescents. Experienced Aboriginal and Torres Strait Islander researchers (AM-D, LJW and TB, all women) conducted the interviews, ranging from 20 min to 45 mins long.

A transcription company transcribed interviews verbatim. Transcripts were not returned to participants for comment or correction as it was not logistically feasible. No participant dropped out or withdrew statements from the study throughout the duration or completion of the project. Completed hard copy forms were kept in a locked cabinet, and digital files were stored on secure servers, accessible only to the project team.

### Data analysis

Analysis commenced after data collection concluded. Data from interviews and field notes were imported into the software NVivo (V.13) to facilitate analysis. Thematic analysis was used through an iterative process led by Aboriginal and Torres Strait Islander investigators (AM-D, TB and LJW) and supported by an experienced non-Indigenous qualitative researcher (KA). First, AM-D, TB and LJW created initial codes by coding three transcripts each, discussing emerging concepts and cross-checking coding for consistency. Codes were generated inductively from the data and deductively using an ecological model of health promotion.^18^ Once codes were agreed on, AM-D created a coding structure reviewed by TB and LJW, followed by a coding summary of the initial three transcripts, which was reviewed by TB, LJW and KA. The codes were finalised and AM-D coded the remaining 15 transcripts. Coding summaries were reviewed by TB, LJW and KA before use in interpreting the data and themes, with the final version written by AM-D.

In line with Rigney’s Indigenist Research approach and as outlined in the Reflexivity section of this paper, it was important that the conceptualisation, data analysis, interpretation of findings and writing were led by Aboriginal and Torres Strait Islander researchers. Under the governance of an Aboriginal and Torres Strait Islander steering committee, these principles were supported and upheld by all researchers on the team. Steering committee meetings were held several times a year throughout the duration of the project. Members provided valuable guidance on the interview guide, ideas for school reciprocity and interpretation of results. Additionally, members had the option to be recognised as coauthors. These principles were particularly important to apply in this subanalysis from a predominantly non-Indigenous key stakeholder group, to appropriately unpack the power dynamics, and structural barriers discussed and to ensure that the focus was on delivering the vaccine to Aboriginal and Torres Strait Islander adolescents.

The application of an ecological model of health for this analysis, allowed for an exploration of factors occurring at the individual level (immunisation providers and school staff knowledge and attitudes), interpersonal level (relationships between families, schools and immunisation providers), institutional level (school policies, programme design, coordination between sectors) and broader system-level contributors (historical and colonial legacies, public health policies). Preliminary findings were reported back to the Aboriginal and Torres Strait Islander Steering Committee and feedback incorporated into the findings.

### Patient and public involvement

As this project involves a public health programme, there was no patient involvement in this study; however, the public were involved in several ways. HPV vaccination was identified as priority in the *National Aboriginal and Torres Strait Islander Cancer Framework,*^22^ which had Aboriginal and Torres Strait Islander leadership and community consultation. This project was conducted with the guidance and collaboration of an Aboriginal and Torres Strait Islander Steering Committee. A key aspect of this involved their participation in interpreting the findings from the interviews with the research team. Recognising their valuable expertise and role as knowledge holders, all steering committee members were offered authorship. Further details of the involvement and governance of Aboriginal and Torres Strait Islander people in this project are provided in online supplemental file 2. We plan to share summaries of the findings of the entire Yarning about HPV vaccination study with participating schools and with individual participants who opted in to receive summaries of the findings.

## Results

We conducted 18 interviews with providers and school staff about their views and experiences engaging with and/or delivering components of the SIP to Aboriginal and Torres Strait Islander adolescents. Participant and school characteristics are provided in table 1.

**Table 1:** Participant and school characteristics

	Number of key informants (%)
Total	18
Gender	
Male	2 (11)
Female	16 (88)
Indigenous identification	
Non-Indigenous	14 (77)
Aboriginal and/or Torres Strait Islander	4 (22)
Age	
Mean	45
SD	8
Median	45
Min	31
Max	57
Remoteness	
Metropolitan	5 (27)
Regional	8 (44)
Remote	5 (28)
School sector	
Catholic	3 (30)
Government	5 (50)
Independent	2 (20)
School size*	
Small	1 (10)
Medium	6 (60)
Large	3 (30)
Role	
Nurse immuniser	7 (38)
Immunisation coordinator	4 (22)
School administration staff	2 (11)
Student support staff	5 (27)
Years in role	
Mean	5.5
Qualifications	
Certificate, diploma	3 (16)
Bachelor degree	7 (38)
Postgraduate degree (bachelor honours degree, graduate cert, grad dip, masters, doctoral)	4 (22)
Missing data	4 (22)

* Small ≤400 students; medium=401–1000 students; large ≥1001 students.

Thematic analysis of stakeholder perspectives identified four key themes: (1) co-ordination of the clinic between schools and programme providers, (2) supporting Aboriginal and Torres Strait Islander families through the vaccination pathway, (3) HPV vaccination resources and (4) COVID-19 disruptions to HPV vaccination programme. Some of the discussions with participants were about the delivery of the programme in general, and some other issues were specific to Aboriginal and Torres Strait Islander adolescents. Four-digit participant identification codes are provided with quotes.

### Co-ordination of the clinic between schools and programme providers

Programme providers highlighted a need for better communication and coordination between the school clinic coordinator/s and the immunisation clinic providers, citing breakdowns in communication as the main reason for misunderstandings of instructions and inconsistent procedures across the vaccination process. This impacted vaccination delivery in several ways, including the scheduling of clinic visits, complying with venue and clinic requirements and timely management of student vaccination records. Providers highlighted the need for further education of school staff about the vaccination pathway via SIP—from consent through to course completion.

### Clinic venue adherence health and safety requirements

Providers noted that adherence to immunisation health and safety requirements by school staff was inconsistent; additionally, schools frequently described challenges in finding appropriate venues for the clinics to meet safety requirements.

… there’s often a big gap between what we request and the reality of where we’re vaccinated [2828, Nurse Immuniser, Metropolitan].

Providers valued being regarded by schools as competent in their role and understanding the safety requirements of their duties during clinic visits.

I wouldn’t go into a teacher’s class and say, “teach it this way and be done by half past nine.” We’re two professional people together, and when I'm doing clinic and when we’re giving vaccines, the safety side is paramount [2828, Nurse Immuniser, Metropolitan].

Some schools had limited space or capacity to accommodate clinic visits. This was usually due to the small size of the school, building and construction happening on school grounds, walking distance to and from the clinic, and limited amenities like a sink to wash hands. As such, schools sometimes had to facilitate clinic visits in non-ideal venues that were not appropriate for post-vaccination recovery.

So, we were having them go across to another classroom which is over a car park; it’s a big car park. They were like someone needs to escort them over in case they faint and that sort of stuff; it was a long way to walk [1563, Immunisation Coordinator, Remote].

So, we generally like a room with an in and out door. We like to have an area for recovery. We like to have – be able to wash our hands. But seldom [do] we get a sink in the room… normally you have to walk over to the toilets [2828, Nurse Immuniser, Metropolitan].

Two programme providers ran clinics in non-air-conditioned rooms and noted that it was too hot for students, especially during the 15 min recovery period. Other features of the clinic set-up also presented challenges, such as limited privacy for students being vaccinated. Some of the room set ups were too small, had no dividers or designated areas where pupils could be separated by those queuing in the line, those being vaccinated, and those waiting in the recovery area.

It’s not the sort of venue for us to be able to spend huge amounts of time with individual pupils. So, if you have a child that is highly anxious, or adamant they don’t want to have it, we do have limited time to give that pupil [2828, Nurse Immuniser, Metropolitan].

Adherence to clinic venue requirements was paramount for nurse immunisers and programme partners to ensure the safety and comfortability of students, who often felt uncomfortable due to a lack of privacy and other non-optimal venue conditions.

### Data sharing

One provider mentioned the challenges of managing student data, particularly for important tasks like confirming vaccination status to link to Medicare records, school transfers or residential address changes and following up on consent forms. They found that some schools were reluctant to provide this information and other basic demographics, even though this is permitted in legislation and communicated in the SIP information booklets provided to the schools.

… my biggest problem is the delay in data entry, and the delay comes from not having the information of students who don’t return the form, and the school’s reluctance to give that to me, even though I do have legislation backing me, saying that I am allowed to ask and receive this information… I also included the Public Health Act, and I still get push back from that… It’s probably just an attitude towards the entire program. Some schools just see it as an inconvenience… It’s good when I have support from the schools and you’re able to achieve things better, but if the school supports it, you notice it in the consent form return rate, like you really do [4156, School Admin Staff, Regional].

### Scheduling

Programme providers were conscious of schools with limited capacity to host the clinic visit due to other school demands or activities taking place on the same day. Many providers explained that clinics visits were often rushed and there was not enough time allocated to spend with each student, especially at larger schools or visits that included students for catch-up sessions.

Some schools, they do want you in and out really quickly because we’re affecting their curriculum they’ve got, and we’re just a nuisance to them sometimes. They don’t want us there with kids fainting and different things [3363, Nurse Immuniser, Regional].

A lot of schools get upset if clinics go beyond a certain period of time, even though we do tell them it’s going to take however long as it’s going to take, there are things outside our control with delays and various diverse events from the immunisation, like there’s nothing we can do about that [4156, School Admin Staff, Regional].

This impacted nervous students, who ideally would have received more time from immunisers to address their questions and alleviate concerns. However, due to time constraints, some students chose not to get vaccinated.

Nurse immunisers juggled multiple tasks, particularly on the time-pressured vaccination day, such as managing the students’ emotions, providing information, answering queries, supervising and caring for students’ postvaccination and contacting parents if a student declined vaccination or if a consent form was not returned. However, understaffing and short time allocation for the vaccination clinics negatively impacted nurses’ ability to carry out these tasks.

To be honest, there isn’t time for us, we’re not allocated time to ring all of the parents, and all of the children that we don’t vaccinate… there could be as many as 20 children who don’t get vaccinated, and they would all be sent out a letter from admin. However, with the smaller special schools, if I've got time, I ring. And I can't speak for my colleagues. We’re not told to do that, we’re told, if you can, great. So I try and make an effort, and today I made an effort [2828, Nurse Immuniser, Metropolitan].

Immunisation providers observed a number of disruptions to the SIP usually due to issues around the scheduling of clinic visits. Some schools were unprepared for visits due to mixing up dates or school staff forgetting on the actual day. Sometimes school events and construction work in the school grounds that coincided with visits also disrupted the programme.

They had been notified many times about the clinic. It was on their calendar, but the school – they had forgotten. Because they were having work done, building work, they had sent a load of pupils offsite for sport. And they were children that were to be vaccinated [2828, Nurse Immuniser, Metropolitan].

### Supporting Aboriginal and Torres Strait Islander families through the vaccination pathway

School staff and programme providers both agreed that connecting Aboriginal and Torres Strait Islander school staff, programme providers or Indigenous community health organisations, with students and their families not long after they have commenced school, is most effective in building rapport and would facilitate engagement in the HPV vaccination programme. Having enough lead time to engage with families was regarded as being beneficial.

…when we're working with Aboriginal and Torres Strait Islander communities, we talk about the three cups of tea; the first time is just the meet and greet, the second time is that you’re now all of a sudden friends, and then the third time is you’re family, we’ll do anything for you. It’s the same situation [with following-up on consent forms]. It needs a longer lead-in time [1662, Student Support Staff, Metropolitan].

One participant reflected that this can be challenging when there is a large cohort of Aboriginal and Torres Strait Islander students, and adequate time and resources are not embedded in the process.

We are sitting at 24 nearly 25 per cent Indigenous now, so the more Indigenous enrolments we have the harder it is with those sorts of things [following up with consent forms]. So having consistency and support absolutely that work in community and with families and with the school is a vital link [1563, Immunisation Coordinator, Remote].

This was particularly apparent in the completion and follow-up of consent forms and supporting Aboriginal and Torres Strait Islander students on vaccination day.

### Completing and returning consent forms

Completion and return of consent forms were highlighted as a major barrier to HPV vaccination. Staff-reported parents feeling overwhelmed by the influx of forms and information sheets at the beginning of the school year. However, this was an issue common to all students attending the school, not just Aboriginal and Torres Strait Islander students.

… something more needs to be done there because it’s not just Indigenous [students who don’t receive the consent form], we have a lot of kids who don’t get it, and for some of the kids, it’s like, “I didn’t bring the form back in time [1172, Immunisation Coordinator, Metropolitan].

Teaching staff and other school staff were usually juggling multiple roles within the school, and involvement in the HPV vaccination clinic was an additional workload. Competing demands were mentioned frequently, with school staff often too busy, understaffed or not allocated enough time to efficiently carry out tasks involved in coordinating the HPV vaccination clinic, such as providing information and education to students and parents who had questions and concerns. As a result, school staff had very little time to follow-up the return of HPV vaccination consent forms.

… I don’t really have a chance to talk to the kids and say, “Have you brought them back, are you bringing them back?” And ask those questions, I never really have time at the beginning of the year to really stop and pay attention to what is going on there” [1172, Immunisation Coordinator, Metropolitan].

If I had a greater timeline, that would be really helpful. The turnaround time was quite short this time, but it would be about engaging the community elders and those health support networks that are already out there and bringing them in to increase the validity [of HPV vaccination]… So any opportunity to bring the community in, then capitalising upon that, but the lead time is probably the biggest thing [1662, Student Support Staff, Metropolitan].

Some providers and school staff followed up on consent forms via home visits, particularly for Aboriginal and Torres Strait Islander students, in small schools or cohorts. Several schools relied on Aboriginal and Torres Strait Islander school staff to follow up on consent forms by organising home visits and check-ins with Aboriginal and Torres Strait Islander students and their families. This method was also utilised to follow up with students who had missed vaccination due to absenteeism, suspension or other circumstances. One school relied on an Aboriginal and Torres Strait Islander community sport organisation to follow up on consent forms with students. The organisation dropped students to and from school and talked with caregivers at home, so forms could be returned sooner. All staff used existing community networks (eg, Aboriginal and Torres Strait Islander health workers) to connect to caregivers to answer any questions or concerns, assist with completing the consent forms and/or collecting the forms. This was beneficial for caregivers with a low literacy level or those who were simply busy.

So that’s the value of having our health worker workforce, they are just amazing, they know their communities [5274, School Nurse, Remote].

Some participants observed that the return rate of consent forms was highest when teachers took strong initiative to follow-up and encourage students to return them. This often occurred during roll call in class or through home visits, but its effectiveness was determined by the teachers’ attitude and their ability to take on this responsibility.

… a lot of teachers are on the ball, and demanding the form comes back. So, we’ve noticed that’s worked a bit better than (in) previous years, we’d just hold a parade, hand out the forms at the parade, [and] hand them into the office. -- We haven’t applied more pressure because there’s a lot of things that we’re asking teachers to do, so we have to value it [SIP], I think more [3276, Immunisation Coordinator, Regional].

One school staff member shared their experience with some Aboriginal and Torres Strait Islander students facing complex living arrangements or family situations. Factors such as disturbances at home and parents working long hours made it challenging for school staff to conduct home visits.

…we also have quite a few single parents. We also have a lot of kids who are having to parent themselves and little brothers and sisters because their parents are working so hard, and they’re going to work at 6, and getting home at 7 o’clock at night [5285, Student Support Staff, Remote].

These living realities caused challenges for parents receiving the consent form and returning the completed form, which could affect HPV vaccination uptake among Aboriginal and Torres Strait Islander adolescents.

Aboriginal and Torres Strait Islander school staff went out of their way using personal resources and time to ensure consent was received. For boarding school students, this could include meeting with caregivers at the airport or train station and hand-delivering/collecting consent forms. Other methods of collecting consent forms for non-boarding schools were pop-up stalls in shopping centres and in community hotspots where families could drop off consent forms or ask questions—reducing the cost of transport and minimising reliance on the adolescent to take home the form, have it signed and return it.

I'm really big on meeting as a team around a young person, the schools can sometimes fall in that trap of, well, you’re referred there, and then you’re referred there, and you’re referred there. But I know that even our parents don't like that, because a lot of our parents’ experience that out there with agencies. So, I say, no, we’re the one-stop shop. We can do X, Y, Z, and go above and beyond, whatever you need. Even transporting for lifts for parents, for kids. And we don’t get a car. It’s our personal car [4874, Student Support Staff, Remote].

Some school staff promoted HPV vaccination and return of consent forms via the school app, school newsletter and school forum pages on social media platforms such as Facebook. They found that many caregivers preferred online communication about vaccination and that this increased overall engagement and return of consent forms. Paper-based consent forms were still emphasised as the preferred method at one rural school, where the network in this region is unreliable.

### On vaccination day

Aboriginal and Torres Strait Islander school staff were aware that their presence in these students’ lives was important and were committed to being a culturally safe figure and/or support person.

It’s not just about learning stuff in the classroom. This is the safe spot. Because we even find after the weekend, every drama that happened at home, kids will divulge after the weekend. So, this is a safe place for them, especially for us too, we must be the safest people on the planet. Because they come to us with all of these things, baggage, first thing on a Monday morning and that sort of thing. So, if they know that the school is not just about books and reading, it’s also around health and wellbeing [4874, Student Support Staff, Remote].

One school staff member highlighted that this engagement should follow through to vaccination day, so that students are met with a familiar face on arriving at the clinic. In particular, having Indigenous student support staff at the clinic is helpful as they are generally perceived as more ‘relaxed’ rather than ‘control figures’ who may punish certain behaviours.

I know with some of the Indigenous kids, they don’t like interacting with admin. Even if they’re not troublemakers and they’re not getting into trouble, just that authority figure, they don’t like sometimes if there’s no good relationship. So having the non-teaching staff present, and the non-admin people I think has been really helpful [1172, Immunisation Coordinator, Metropolitan].

The presence of Indigenous and non-Indigenous teacher aids, chaplains and school nurses at the clinic on vaccination day was common. Both school staff and programme providers felt that it was important for Aboriginal and Torres Strait Islander students to see a familiar face, especially those students who presented as anxious, had a fear of needles or were prone to fainting. The presence of these staff provided more opportunities to check-in with Aboriginal and/or Torres Strait Islander students who were second-guessing receiving the HPV vaccination postconsent and allowed them to feel more comfortable with the process.

### HPV vaccination resources for Aboriginal and Torres Strait Islander families

#### Health literacy and education among caregivers

Participants discussed how the lack of access to culturally appropriate information and resources coupled with complex consent processes influenced informed decision-making among Indigenous caregivers. Language barriers and diverse literacy levels meant that some Aboriginal and Torres Strait Islander caregivers had a limited understanding of the consent form and were not always sure what they were signing or consenting to. Additionally, the readability of the form was considered too long, with too much text.

…some families don’t have Internet access, and some are illiterate, you know what I mean? They don’t understand all this writing and they may just sign and don’t know what they’re signing for [1737, Student Support Staff, Remote].

As mentioned earlier, Indigenous support staff typically assisted Aboriginal and Torres Strait Islander students and their caregivers with filling out consent forms. School staff believed that community health education about HPV vaccination would help increase uptake.

I think it is still educating parents about the vaccine, because sometimes they’re signing consent forms and they’re not even really aware of what they’ve signed. When things have gone wrong, like, they’ve got the wrong injection in the past, when you ring the parents, they said, “Oh, I didn’t even know they were getting that vaccine,” or things like that. Yes, educating parents [3363, Nurse Immuniser, Regional].

They didn't have an understanding around what it was. So, I guess it’s almost facilitating those conversations at home around why it’s important. And as part of health checks and everything else, they know about healthy eating and everything like that, but where does immunisation sit and specifically HPV sit within that? [9373, Student Support Staff, Metropolitan].

#### Culturally appropriate resources and information

At some schools, information and education about HPV vaccination were provided through health promotion talks by QLD Health staff, and teacher-led talks or initiatives to distribute information widely.

I created just a little pamphlet, flyer thing for the school to blast to the caregivers about sending their consent forms and that they can send them electronically to the generic school immunisation program email, as well as the school, which is something I will try and implement in the future [4156, School Admin Staff, Regional].

Most schools did not provide any extra resources or information packs outside of the standard government HPV resources, but agreed, at times, that this was not enough information for students and caregivers. Some school staff agreed that the resources currently used at their school were not considered culturally appropriate for Aboriginal and Torres Strait Islander audiences. Some schools provided some extra information to students and caregivers, but this was prompted by school staff who noticed a low return of consent forms or received questions from parents and students about the vaccine. Some staff shared YouTube videos with students and personally called up parents to answer their questions and concerns.

School staff reported a need for further education and/or training for conversations around HPV vaccination with students and caregivers. Accordingly, staff were generally proactive in keeping up to date or doing their own research to feel more informed and competent.

Look, I try and read up as much as I can. And I will say to them, okay, hang on, just let me just go back over and refresh [5285, Student Support Staff, Remote].

#### COVID-19 disruptions to SIP

The COVID-19 pandemic played a significant role in the disruption of the SIP across QLD schools. Clinic visits were postponed due to school shutdowns, lockdowns and limited availability of nurse immunisers. Providers aimed to send consent forms to schools early in the year and facilitate clinics as early as possible in case lockdowns occurred later in the year.

COVID-19 restrictions and public health orders made it even more challenging for some schools to choose suitable venues for the vaccination clinic, where social distancing could be maintained.

We got our first lot of needles just as corona was kicking in. But we had to then spread them [students], so we couldn’t do this. We had them in the big hall, so that we could put space between them all, and chairs were further apart. So, it was a bit of a logistical thing, but we still made it happen. And then we were all back by September, so social distancing was accepted then. Where it was a weird thing. We’re thinking of each other… spread out beforehand it hadn’t had much of a meaning [5285, Student Support Staff, Remote].

Accordingly, some immunisers had to modify their usual approaches to comfort apprehensive students.

Sometimes people do want to hold hands. This is another drama with COVID actually, that you don’t tend to want to be offering to hold hands. But if I thought it was going to tip them [student] over - - - I would say do you want to hold hands… then we’d all just wash our hands after. I'm wearing a mask still at the moment, because you can't social distance. As a general rule, we’re trying not to touch [2828_Nurse Immuniser, Metropolitan].

Providers noted that, following the rollout of the COVID-19 vaccine, some students were getting vaccinated at their General Practice or community clinic and were unable to receive the HPV vaccine via the SIP as they had not completed the ATAGI-recommended 7-day waiting period. There was also confusion among some students who thought they had already received the HPV vaccine or thought they were receiving the COVID-19 vaccine at school.

I think it’s made it much harder for everyone. I think it’s messier. I think we've not been able to vaccinate quite a few children that we would have liked to. And we have been trying to go back and do some of those, or get them into the catch-up clinic, but it is messier than normal [2828, Nurse Immuniser, Metropolitan].

Some kids, I think, I’m not sure whether the message gets a little distorted between school and home, but some parents thought that we were giving COVID vaccination [5274, Nurse Immuniser, Remote].

Some providers and school staff had to complete extra work to double check if caregivers were still consenting to the HPV vaccine, due to heightened public dialogue around the COVID-19 vaccine at the time and polarised public attitudes. This confusion resulted in some students not receiving the HPV vaccine during the school clinic.

… there’s also a risk that parents will withdraw consent. So, having to really make sure, to do triple checking… because COVID has impacted [processes]: has the student gone elsewhere? Is it up on AIR [Australian Immunisation Registry]? Has a parent contacted us? So, making sure that going out there and vaccinating, that the parents still give consent, and they’ve already signed the form [8092, Nurse Immuniser, Metropolitan].

I would ring the parent and just say, “What do you want to do? Do you want to defer that [vaccine] and them [adolescent] have their vaccine today, or would you like me not to vaccinate today, then to have the COVID [vaccine], and then you have this done at the GP?” Because it’s the parents’ choice which vaccine they put first [2828, Nurse Immuniser, Metropolitan].

## Discussion

This study aimed to understand the perspectives of immunisation programme providers and school staff (key stakeholders) involved in, and responsible for, delivering the SIP adolescents in Queensland, with a focus on HPV vaccination uptake among Aboriginal and Torres Strait Islander adolescents. Key stakeholders in our study identified four key themes that were important to HPV vaccination delivery in general and for Aboriginal and Torres Strait Islander adolescents: (1) the coordination of the vaccination clinic between the school and programme providers; (2) supporting Aboriginal and Torres Strait Islander adolescents and their families through the vaccination pathway; (3) HPV vaccination resources and (4) the disruptions to HPV vaccination programme from COVID-19. Stakeholders in this study highlighted the complexities navigating an SIP as it traverses health and education sectors, identifying the challenges involved in coordinating and scheduling, roles and responsibilities, communication and logistics between schools and school staff, programme providers and immunisers, parents and caregivers, adolescents and the consent processes in an evolving COVID-19 and policy context. Schools have long been effective settings for delivering large-scale vaccination programmes to adolescents, both in Australia and internationally.^3 23^ This study gathers stakeholder perspectives regarding key factors that influence implementation and significantly impact Aboriginal and Torres Strait Islander adolescents and achieving equity.

### Supporting staff and systems for effective coordination of school-based vaccination clinics

The importance of supporting teachers and the education workforce involved in delivering vaccination is a key learning from HPV implementation globally^24^,^26^ and was an important finding from this study. We found variation in capacity of staff to support HPV vaccination pending different characteristics such as school support, smaller student cohort, early coordination in the school year which enabled teachers’ time to connect with carers to build rapport, follow-up on consent forms through home visits and spend more time discussing HPV information with students, allowing them to answer questions more effectively and are reflective of previous findings.^27^ In line with current findings,^3^ strong initiative and efforts from both school and delivery staff were seen to be the main drivers of high return of consent forms, and increased uptake; however, stakeholders reported the negative downstream impacts of reduced staff capacity on adolescents receiving their vaccination. Schools relied on Aboriginal and Torres Strait Islander school staff to follow up consent forms with Aboriginal and Torres Strait Islanders students. Despite staff capacity, Aboriginal and Torres Strait Islander school staff and other student support staff were self-driven and committed beyond their usual roles and responsibilities, doing many home visits to follow up on consent forms or assist caregivers with filling out consent forms. School staff must continue to be supported at all phases of the vaccination pathway through ‘whole school commitment and involvement’^3^ ^p. 9^ and systems must be responsive to these needs.

Programme providers’ effectiveness relies heavily on schools’ adherence to venue and clinic setup guidelines and allowing sufficient clinic time, as noted in prior studies.^3 28^ Schools should aim to follow room requirements to ensure the safety and privacy of students during vaccination. Our findings suggest that school staff supporting the programme would benefit from additional education on SIP guidelines to meet health and safety standards, including time allowances, as this has been an ongoing issue.^27^ Adolescents in the SIP should be afforded the same baseline standards as any other vaccination clinic.

To lessen the load on school staff, particularly Aboriginal and Torres Strait Islander staff balancing cultural and organisational roles, schools could benefit from clearer role descriptions, increased support and designated staff for consent follow-ups. Programme success depends on the capacity of both school staff and providers and ultimately this means that schools and Queensland’s education system must place value on, and devote resources to, supporting the SIP as an important public health programme.

### Engaging and supporting Aboriginal and Torres Strait Islander adolescents and caregivers

#### The role of Aboriginal and Torres Strait Islander school staff

Our findings highlight that connecting with Aboriginal and Torres Strait Islander adolescents and carers via Aboriginal and Torres Strait Islander school staff is essential. Aboriginal and Torres Strait Islander school staff often have existing networks and ties to the community and are fundamental in supporting Aboriginal and Torres Strait Islander students and caregivers with informed decision-making, return of consent forms and supporting adolescents’ well-being. Aboriginal and Torres Strait Islander education and staff in schools have been described as critical to the teaching and learning experiences of Aboriginal and Torres Strait Islander students and that their role is both multifaceted and undervalued.^27^ The role of Aboriginal and Torres Strait Islander school staff represents a missing element of the cultural interface that manifests in school settings. The cultural interface is a framework that explains the cultural interactions and exchanges between Indigenous knowledges and Western knowledges and seeks to bridge the epistemological gap through meaningful engagement from all parties.^29^ The role of Aboriginal and Torres Strait Islander staff was as *Armour et al* describe ‘the cultural broker between the school and local Aboriginal and Torres Strait Islander community’.^27^ Our study highlights that the role of Aboriginal and Torres Strait Islander staff is not just critical in the education but also in the context of delivering HPV vaccination in schools and must be supported to facilitate meaningful engagement with Aboriginal and Torres Strait Islander students. They are the ‘cultural bridge’ between the school and community and prioritising these relationships is essential to maintaining trust, reliability and confidence in schools and providers.

However, schools need to implement measures to ensure that Aboriginal and Torres Strait Islander school staff are not overburdened or undervalued and are adequately supported to deliver such a crucial component of the SIP. Similarly, government accountability and greater investment are critical to ensure that schools are adequately resourced, and that all school staff are provided appropriate training—in terms of cultural safety and the logistical delivery of the SIP. There is also a need to recognise how schools have not historically been considered a culturally safe or inclusive environment by many Aboriginal and Torres Strait Islander peoples,^27^ and this continues to influence how Indigenous families engage with the education system. It is important that responsibility of others is not absolved, and schools and communities are not viewed as separate entities^30^ and that equitable HPV vaccination coverage is everyone’s business. Accordingly, schools with a higher percentage of Aboriginal and Torres Strait Islander students in their cohort should consider having a proportionate amount of Aboriginal and Torres Strait Islander school staff or other school support staff to perform these responsibilities and maintain relationships.^29^ Aboriginal and Torres Strait Islander school staff should also be supported to have culturally sensitive and safe conversations about HPV vaccination with Aboriginal and Torres Strait Islander adolescents and caregivers, as they are best placed to facilitate matters of this nature.

Furthermore, partnerships between schools and local Aboriginal and Torres Strait Islander community health organisations should be encouraged as they can be useful in reaching adolescents and families who are already linked in with them. Both Indigenous and non-Indigenous school staff need greater support to develop and maintain strong professional relationships with Aboriginal and Torres Strait Islander communities as well as with and Aboriginal Community Controlled Health Organisations and AMSs.^12^

Clinic visits can be further supported by the presence of Aboriginal and/or Torres Strait Islander school staff and nurse immunisers, or health workers especially from existing community health clinics, who can be a familiar or supportive figure for adolescents. Aboriginal and Torres Strait Islander students should not have to rely on the goodwill of school staff to go beyond their work hours and normal scope of practice to carry out these duties.

#### Flexible methods of consent

School staff reported using varied and multiple methods to follow-up or confirm consent however consistently identified that paper-based forms were resource intensive to follow up, although in some settings necessary due to internet coverage. We recommend that flexible models of providing consent, including alternatives to conventional paper-based methods, are implemented to improve the return rate of consent forms. These models should help to address the structural obstacles Aboriginal and Torres Strait Islander families may face with paper-based forms and the additional follow-up that Aboriginal and Torres Strait Islander school staff perform to gain signed consent forms for students including those at boarding school. These must be driven by localised strategies that suit each community’s needs.

#### Culturally sensitive resources

Overall, most school staff and providers emphasised a greater need for informative and educational resources beyond the standard government HPV vaccination brochures. Research has found that further development of HPV vaccination resources in a range of formats for Aboriginal and Torres Strait Islander audiences is needed.^31^ Our findings, and others,^32 33^ indicate a need for resources to be more effectively embedded in the delivery of the SIP programme to support school and programme provider staff to deliver information systematically and effectively.

#### COVID-19 pandemic and learnings for future disruptions

HPV vaccination coverage was steadily increasing before the pandemic but has since decreased among all demographic groups including Aboriginal and Torres Strait Islander adolescents.^2 7^ The COVID-19 pandemic and its associated public health measures including lockdowns, school closures and social distancing significantly disrupted school programmes, as reflected in the immunisation data from this period.^7 8 32^ Subsequently, one-dose HPV vaccine rates for Aboriginal and Torres Strait Islander girls and boys were up to 3.1% lower in 2022 than in 2021 and were also lower when compared with adolescents overall.^32^ In 2022, 83% and 78.1% of Indigenous boys and girls, respectively, received one dose of HPV vaccine by age 15, decreasing in 2023 for both groups (80.9% vs 75%), with even lower coverage observed by jurisdiction of residence.^7 8^ Stakeholders also reported on the major disruptions to the SIP caused by the pandemic. They talked to the additional workloads navigating public health measures, for example, social distancing and school closures, but also the polarising public commentary regarding the COVID-19 vaccine, which has been reported similarly elsewhere.^28 33^ The rollout of COVID-19 vaccinations also impacted students’ eligibility to receive the HPV vaccination at the school clinic. Accelerated catch-up efforts and strategies to minimise both long and short-term impacts of SIP disruptions are crucial, as delays would result in further preventable cervical cancer cases in future.^34^

Our findings reflect those elsewhere^35^ regarding the need for flexible immunisation clinics that are adaptable to mitigate such challenges in the future. The ecological model applied to our analysis illustrates the importance of not only cross-sectoral strategies that address school-level operational issues but also structural inequities that contribute to lower HPV vaccine uptake and acceptance among Aboriginal and Torres Strait Islander adolescents and their caregivers. Some of these strategies include clear and consistent messaging to ensure students and parents are well-informed, and more regular communication to programme providers and schools in evolving and changing circumstances. Additional care and planning may be needed, including setting up extra vaccination clinics to maintain adequate coverage and minimise disruption to education during similar events.

### Strengths and limitations

This study was guided by Nurungga Professor Rigney’s Indigenist research approach^17^ and as such was led and guided by Aboriginal and Torres Strait Islander researchers, a steering committee and prioritised the knowing and experiences of Aboriginal and Torres Strait Islander peoples. To the best of our knowledge, there are currently no other published studies to date which examine stakeholder perspectives of HPV vaccine uptake among Aboriginal and Torres Strait Islander adolescents and caregivers via the SIP.

While the study was limited to Queensland, a similar methodology can be employed to conduct further external studies or adapt these recommendations to other States and Territories. We recognise that there are diverse school settings and so the challenges presented, and potential solutions will vary. Governance structures and funding allocation also differ between states and territories, so recommendations must be tailored to the specific needs and capacities of different communities. As such, additional research in other states is required to capture the perspectives and experiences of school staff and providers, and how uptake is impacted in other cohorts of Aboriginal and Torres Strait Islander adolescents and their caregivers.

As noted in the Recruitment and Participants section, Principals and Teachers were invited to participate in the study. We were unable to state how many, due to the small sample size being potentially identifying. Therefore, they are represented by the category ‘school staff’. As qualitative data strives for richness and depth of data rather than representativeness achieved through large sample sizes, the sample size adequately captures a wide range of relevant views.

Data collection for this study took place through 2020 and 2021 at the height of the COVID-19 pandemic, which caused major international, national, state, local and individual impacts to day-to-day life. The COVID-19 vaccine rollout in 2021 received major media attention and contributed to public and online debate around its safety and efficacy.^15^ This context provided the research team with real-time insights from key stakeholders about the perception of the COVID-19 vaccine and the HPV vaccine among not only adolescents and caregivers during a polarising period, but also their perspectives on navigating the SIP during a particularly difficult time. The COVID-19 pandemic interrupted various research activities, particularly some interviews with school staff and programme providers that were initially scheduled during clinic visits. As such, findings from this period do not represent a ‘normal day’, however, still provide insight into how students, schools, providers and systems were impacted by a phenomenon that, in future, could become more frequent.

### Recommendations

We provide a number of recommendations that support the current literature on stakeholders’ perspectives of HPV vaccination of Indigenous adolescents. Recommendations previously identified in an impact evaluation of the national HPV vaccination programme in Australia and international literature, align with the findings^3 15 24^ raised by stakeholders in this study. As previously mentioned, further research is recommended to explore state-specific differences in the school-based HPV vaccination programme. The following recommendations can be highlighted to ensure ongoing, improved HPV vaccination for Aboriginal and Torres Strait Islander students in the SIP:

- Partnerships between schools and local Aboriginal and Torres Strait Islander community health organisations should be encouraged as they can be useful in reaching adolescents and families who are already linked in with them.
- Equipping both teaching and non-teaching staff with the most up-to-date information and resources about HPV vaccination is vital for teachers to feel confident answering questions and assisting students and caregivers with their concerns.
- Ensure sufficient school staff and programme provider capacity to support the HPV vaccination programme in all aspects of promotion, follow-up and completion.
- Ensure school staff, nurse immunisers and/or health workers who are familiar to and trusted by Aboriginal and Torres Strait Islander adolescents are present on vaccination day to provide additional support for the clinic.
- Develop additional culturally appropriate resources to support school staff and programme providers to provide information and education to Aboriginal and Torres Strait Islander families.
- Implement flexible models of consent, guided by the needs of the local community.
- Ensure additional catch-up clinics are scheduled to avoid missed opportunities to vaccinate as a result of the one-dose schedule.

## Conclusion

School-based immunisation clinics are among the most effective settings for maximising HPV vaccine coverage among adolescent populations. This study contributes stakeholder insights specifically relating to HPV vaccination uptake among Aboriginal and Torres Strait Islander adolescents, who are identified as a priority population in Australia’s National Strategy to Eliminate Cervical Cancer.^10^ Cervical cancer elimination requires concerted efforts from all stakeholders to achieve Australia’s elimination targets. Implementation of the recommendations for policy and practice will support programme providers and school staff in their roles in SIP and contribute to achieving equity in cervical cancer outcomes among Aboriginal and Torres Strait Islander peoples in Australia.

## Supplementary material

10.1136/bmjopen-2024-097518 online supplemental file 1

10.1136/bmjopen-2024-097518 online supplemental file 2

10.1136/bmjopen-2024-097518 online supplemental file 3

## Data Availability

All data relevant to the study are included in the article or uploaded as supplementary information.
