# Supplementary material for: Stakeholder perspectives on HPV vaccination uptake among Aboriginal and Torres Strait Islander adolescents via the school immunisation programmes in Queensland: a qualitative study
Source: BMJ Open. 2025 Jun 4;15(6):e097518. doi: 10.1136/bmjopen-2024-097518 (PMC12142122; doi:10.1136/bmjopen-2024-097518)
Supplement: online supplemental file 1 [file bmjopen-15-6-s001.docx]

**Supplementary File 1 –** *Stakeholder perspectives on HPV vaccination uptake among Aboriginal and Torres Strait Islander adolescents via the School Immunisation Program in Queensland*

**Consolidated criteria for reporting qualitative studies (COREQ): 32-item checklist (Tong et al., 2007)**

| **No** | **Item** | **Guide questions/description** | **Relevant section of text and elaboration if necessary** |
| --- | --- | --- | --- |
| 1. | Interviewer/facilitator | Which author/s conducted the interview or focus group? | Third paragraph under “Data collection” |
| 2. | Credentials | What were the researcher's credentials? *E.g. PhD, MD* | Formal credentials not reported but research team experience summarised under “Reflexivity” |
| 3. | Occupation | What was their occupation at the time of the study? | Researcher occupations and roles described under “Reflexivity” |
| 4. | Gender | Was the researcher male or female? | Third paragraph under “Data collection” |
| 5. | Experience and training | What experience or training did the researcher have? | Research team experience listed under “Reflexivity”. |
| 6. | Relationship established | Was a relationship established prior to study commencement? | Third paragraph under “Data collection” |
| 7. | Participant knowledge of the interviewer | What did the participants know about the researcher? e*.g. personal goals, reasons for doing the research* | Third paragraph under “Data collection” |
| 8. | Interviewer characteristics | What characteristics were reported about the interviewer/facilitator? e.g. *Bias, assumptions, reasons and interests in the research topic* | Under “Reflexivity” |
| 9. | Methodological orientation and Theory | What methodological orientation was stated to underpin the study? *e.g. grounded theory, discourse analysis, ethnography, phenomenology, content analysis* | Described under “Study approach” and “Data analysis”. Further detail reported in the Protocol paper (Whop et al., 2021) |
| 10. | Sampling | How were participants selected? *e.g. purposive, convenience, consecutive, snowball* | Second paragraph in “Recruitment and Participants” |
| 11. | Method of approach | How were participants approached? e*.g. face-to-face, telephone, mail, email* | Second paragraph in “Recruitment and Participants” |
| 12. | Sample size | How many participants were in the study? | See first paragraph of “Results” section and Table 1 in “Results” |
| 13. | Non-participation | How many people refused to participate or dropped out? Reasons? | Last paragraph of “Data collection” |
| 14. | Setting of data collection | Where was the data collected? e*.g. home, clinic, workplace* | First paragraph of “Data collection” |
| 15. | Presence of non-participants | Was anyone else present besides the participants and researchers? | First paragraph of “Data collection” |
| 16. | Description of sample | What are the important characteristics of the sample? *e.g. demographic data, date* | See Table 1 in “Results” |
| 17. | Interview guide | Were questions, prompts, guides provided by the authors? Was it pilot tested? | Key topics described in third paragraph under “Data collection”. |
| 18. | Repeat interviews | Were repeat interviews carried out? If yes, how many? | N/A |
| 19. | Audio/visual recording | Did the research use audio or visual recording to collect the data? | First paragraph of “Data collection” |
| 20. | Field notes | Were field notes made during and/or after the interview or focus group? | First paragraph under “Data collection” |
| 21. | Duration | What was the duration of the interviews or focus group? | Third paragraph under “Data collection” |
| 22. | Data saturation | Was data saturation discussed? | Second paragraph under “recruitment and participants” |
| 23. | Transcripts returned | Were transcripts returned to participants for comment and/or correction? | Last paragraph under “Data collection” |
| 24. | Number of data coders | How many data coders coded the data? | “Data analysis” |
| 25. | Description of the coding tree | Did authors provide a description of the coding tree? | Not reported |
| 26. | Derivation of themes | Were themes identified in advance or derived from the data? | “Data analysis” |
| 27. | Software | What software, if applicable, was used to manage the data? | “Data analysis” |
| 28. | Participant checking | Did participants provide feedback on the findings? | Last paragraph under “Data collection” |
| 29. | Quotations presented | Were participant quotations presented to illustrate the themes / findings? Was each quotation identified? e*.g. participant number* | “Results” and participant number, participant type and geographical information provided for each quote. |
| 30. | Data and findings consistent | Was there consistency between the data presented and the findings? | “Results” |
| 31. | Clarity of major themes | Were major themes clearly presented in the findings? | “Results” |
| 32. | Clarity of minor themes | Is there a description of diverse cases or discussion of minor themes? | “Results” |
