# Supplementary material for: Stakeholder perspectives on HPV vaccination uptake among Aboriginal and Torres Strait Islander adolescents via the school immunisation programmes in Queensland: a qualitative study
Source: BMJ Open. 2025 Jun 4;15(6):e097518. doi: 10.1136/bmjopen-2024-097518 (PMC12142122; doi:10.1136/bmjopen-2024-097518)
Supplement: online supplemental file 2 [file bmjopen-15-6-s002.docx]

**Supplementary File 2** – Stakeholder perspectives on HPV vaccination uptake among Aboriginal and Torres Strait Islander adolescents via the School Immunisation Program in Queensland Supplementary

**CONSIDER statement - Checklist of items to include when reporting health research involving Indigenous Peoples (Huria et al., 2019).**

| **CONSIDER statement checklist item** | **Section where item is described in manuscript and elaboration if necessary** |  |
| --- | --- | --- |
| **Governance** |  |  |
| 1. Describe partnership agreements between the research institution and Indigenous-governing organization for the research, (e.g., Informal agreements through to MOU (Memorandum of Understanding) or MOA (Memorandum of Agreement)). | N/A - There was no relevant Indigenous-governing organization for the research with whom to form a partnership agreement.  However, an Aboriginal and Torres Strait Islander Steering Group was formed for the project with Terms of Reference for the scope and purpose of the group as described in the Governance section, under Methods |  |
| 2. Describe accountability and review mechanisms within the partnership agreement that addresses harm minimization. | N/A - There was no relevant Indigenous-governing organization for the research  The Steering Committee Terms of Reference contained procedures for concerns and complaints to be directed to the Project Leader A/Prof Lisa Whop or to an ethics committee. |  |
| 3. Specify how the research partnership agreement includes protection of Indigenous intellectual property and knowledge arising from the research, including financial and intellectual benefits generated (e.g., development of traditional medicines for commercial purposes or supporting the Indigenous community to develop commercialization proposals generated from the research). | N/A as no research partnership agreement. However, we have included the statement  “Informants retain ownership of Aboriginal and Torres Strait Islander knowledge and cultural heritage” in the “Acknowledgements” section and also on participant Consent forms form the project. This was a requirement of the HREC of the Northern Territory Department of Health and Menzies School of Health Research approval. |  |
| **Prioritization** |  |  |
| 4. Explain how the research aims emerged from priorities identified by either Indigenous stakeholders, governing bodies, funders, non-government organization(s), stakeholders, consumers, and empirical evidence | Under “Study Approach”.  The “Introduction” describes the National Strategy for the Elimination of Cervical Cancer in Australia in which Aboriginal and Torres Strait Islander people are identified as a priority population (paragraph 4). Furthermore, the Introduction describes the limited research regarding stakeholders’ perspectives on Aboriginal and Torres Strait Islander people’s HPV vaccination uptake (paragraph 5). Finally, further rationale for the research aims is described in the Protocol paper for this project (Whop et al., 2021). |  |
| **Relationships (Indigenous stakeholders/participants and Research team)** |  |  |
| 5. Specify measures that adhere and honor Indigenous ethical guidelines, processes, and approvals for all relevant Indigenous stakeholders, recognizing that multiple Indigenous partners may be involved, e.g., Indigenous ethics committee approval, regional/national ethics approval processes. | Governance processes outlined under “Governance”  Ethics Approvals outlined under “Ethics approval and consent to participate”, which includes several Aboriginal and Torres Strait Islander-specific HREC approvals. |  |
| 6. Report how Indigenous stakeholders were involved in the research processes (i.e., research design, funding, implementation, analysis, dissemination/recruitment). | Involvement and contributions of Indigenous Chief Investigators provided under “Reflexivity” under “Methods”  Governance processes outlined under “Governance” – outlines the role of Aboriginal and Torres Strait Islander people providing guidance for the study  Indigenist research approach outlined under “Study Approach”  Involvement of Aboriginal and Torres Strait Islander people in data collection and analysis described under “Data collection” and “Data Analysis” |  |
| 7. Describe the expertise of the research team in Indigenous health and research. | Described in “Reflexivity” section including details of Aboriginal and Torres Strait Islander and non-Indigenous researchers’ expertise |  |
| **Methodologies** |  |  |
| 8. Describe the methodological approach of the research including a rationale of methods used and implication for Indigenous stakeholders, e.g., privacy and confidentiality (individual and collective) | Under “Study approach”, “Data collection” |  |
| 9. Describe how the research methodology incorporated consideration of the physical, social, economic and cultural environment of the participants and prospective participants. (e.g., impacts of colonization, racism, and social justice). As well as Indigenous worldviews. | Under “Study approach”, which includes a description of the socio-ecological model for health promotion which scaffolded the study approach and analysis  Involvement of Aboriginal and Torres Strait Islander people in data collection and analysis described under “Data collection” and “Data Analysis” |  |
| **Participation** |  |  |
| 10. Specify how individual and collective consent was sought to conduct future analysis on collected samples and data (e.g., additional secondary analyses; third-parties accessing samples (genetic, tissue, blood) for further analyses). | N/A future analysis not planned |  |
| 11. Described how the resource demands (current and future) placed on Indigenous participants and communities involved in the research were identified and agreed upon including any resourcing for participation, knowledge, and expertise | As described under “Recruitment and participants” potential participants were initially identified by a School Representative. Following this, the research team held responsibility for any action, reducing burden on Indigenous participants and communities.  Steering Committee members were remunerated for their time; see “Governance” |  |
| 12. Specify how biological tissue and other samples including data were stored, explaining the processes of removal from traditional lands, if done, and of disposal. | No tissue samples collected. Procedures for storage of hardcopy and digital data described in last paragraph of “Data collection” |  |
| **Capacity** |  |  |
| 13. Explain how the research supported the development and maintenance of Indigenous research capacity (e.g., specific funding of Indigenous researchers). | Specific funding for Tamara Butler, an Aboriginal post-doctoral researcher, was provided by the grant. Described under “Funding”. |  |
| 14. Discuss how the research team undertook professional development opportunities to develop the capacity to partner with Indigenous stakeholders? | As described in the second paragraph under “Recruitment and participants”, local researchers advised the research team on appropriate protocol and practices. |  |
| **Analysis and interpretation** |  |  |
| 15. Specify how the research analysis and reporting supported critical inquiry and a strength-based approach that was inclusive of Indigenous values. | Use of Indigenist research approach and leadership of Aboriginal and Torres Strait Islander researchers in study design, analysis and interpretation described in “Study Approach”, “Reflexivity” and “Data analysis”. Furthermore, the use of the socio-ecological model for health promotion (see “study approach”) ensured a focus on structural and systemic factors that may support HPV vaccination.  The Aboriginal and Torres Strait Islander Steering Committee also provided advice on the interpretation of the findings to support critical inquiry and a strengths-based approach. See “Governance” |  |
| **Dissemination** |  |  |
| 16. Describe the dissemination of the research findings to relevant Indigenous governing bodies and peoples. | The findings were checked and shared with Aboriginal and Torres Strait Islander Steering Committee. See “Governance” and last paragraph of “data analysis” and the Patient and Public Involvement statement.  . |  |
| 17. Discuss the process for knowledge translation and implementation to support Indigenous advancement (e.g., research capacity, policy, investment). | This information is not described in the paper due to word limit constraints but provided here: We have presented the findings of this paper at major conferences such as the Eliminating Cervical Cancer conference and International Papillomavirus Conference, and Pacific Region Indigenous Doctors Congress. We will write and present policy briefs to relevant peak Indigenous bodies such as Queensland Aboriginal and Islander Health Council and Queensland Health. |  |
