# Supplementary material for: Stakeholder perspectives on HPV vaccination uptake among Aboriginal and Torres Strait Islander adolescents via the school immunisation programmes in Queensland: a qualitative study
Source: BMJ Open. 2025 Jun 4;15(6):e097518. doi: 10.1136/bmjopen-2024-097518 (PMC12142122; doi:10.1136/bmjopen-2024-097518)
Supplement: online supplemental file 3 [file bmjopen-15-6-s003.docx]

**Yarning guide**

**State-level key informants/Immunisation program partners**

**YARNING GUIDE**

PRESS START ON THE RECORDER NOW

Just for the recording - this is [interviewer name] and I’m with [participant name]. It’s [date] and we’re at [school name OR location]. That is just to help me find our yarn later - your name won’t be reported anywhere. Nothing you say will be traced back to you.

You do not have to answer any questions you do not want to. Just tell me you want to skip the question. You are free to stop at any time without having to give a reason.

There are no right or wrong answers. We are just interested in your experiences.

If online or over the phone: Just for the recording can I confirm that you are happy to take part in this yarn?

Your role

I’d like to understand how the HPV vaccination delivery process happens at *[school/public health unit/other relevant level of focus*] and your role in it. To help with this, throughout our interview I might check in with you to clarify key milestones, people involved and any barriers and challenges along the way.

To get us started, can you tell me about your role? How long have you been working in this role?

Can you please describe how the school-based immunisation program works and describe your role and responsibilities in relation to the Queensland school-based HPV vaccination program at [*school/public health unit/department*].

Policy

What are the major relevant policies in this space?

Are there any specific policies in place to support Aboriginal and Torres Strait Islander adolescents to receive HPV vaccination? If so, can you please describe them?

Information and communication

Are there any aspects of your role that involve providing information about the program or communicating about it to others? If so, what kind of information and who do you provide it to?

Are there any specific information or resources relating to vaccination of Aboriginal and Torres Strait Islander students?

Data

Describe the data management procedures involved in the HPV vaccination program. What data is collected, where is it stored, who is responsible for data management, quality and timeliness?

Are there regular analysis and reporting conducted with this data?

Is there any analysis of Aboriginal and Torres Strait Islander adolescents’ uptake and completion?

What data is provided to immunisation service providers? For example, class lists and line lists for follow-up?

Are there any differences in uptake of HPV vaccination compared to dTpa? If so, why do you think that is?

Pandemic

Was there anything different about how the HPV vaccination was delivered in 2020 compared to 2019 due to the COVID-19 pandemic?

Describe any impacts the COVID-19 pandemic had on the delivery of HPV vaccination at this [school/public health unit/other]

Improving HPV vaccination

Are you required to meet specific vaccination targets? What are these and how they determined?

How is the performance of program understood or evaluated in this HHS?

Does the HPV vaccination program meet the needs of all students? Why do you say that?

Do you feel that Aboriginal and Torres Strait Islander students are supported in the HPV vaccination program? Describe anything you think is working well in supporting Aboriginal and Torres Strait Islander students to have the HPV Vaccination.

Why do you think these things are working well?

Is there anything you would change about how the HPV vaccination program to better support Aboriginal and Torres Strait Islander students? Why would you change these things?

What would you do instead/what would work better?

To what extent would you say that equitable HPV vaccination is the responsibility of your role?

If you were asked to ensure at least 90% vaccination coverage was achieved for all students, how would you go about this? What would you need to achieve this? *E.g., support, policy, resources etc.*

Conclusion

Is there anything else you want to say about HPV vaccinations?

**WRAPPING UP**

That’s it for our yarn today. I’m stopping the recording now.

PRESS STOP ON THE RECORDER NOW

**Yarning guide**

**Local Key Informants/HPV vaccination program delivery staff**

**(including school staff and staff from other organisations)**

**YARNING GUIDE**

PRESS START ON THE RECORDER NOW

Just for the recording - this is [interviewer name] and I’m with [participant name]. It’s [date] and we’re at [school name OR location]. That is just to help me find our yarn later - your name won’t be reported anywhere. Nothing you say will be traced back to you.

You do not have to answer any questions you do not want to. Just tell me you want to skip the question. You are free to stop at any time without having to give a reason.

There are no right or wrong answers. We are just interested in your experiences.

Your role

Can you tell me a bit about your role and responsibilities are in relation to the HPV vaccination program at [school] and/or Aboriginal and Torres Strait Islander students?

How long have you been working in this role?

Describe your role on a day-to-day basis? What does a typical day look like?

I’d like to understand how the HPV vaccination delivery process happens at this school and your role in it.

Information

Does your role involve providing any information about HPV vaccination? This might include to [*school*] staff, parents/caregivers, students? If yes, tell me a bit about this information - where does it come from, who creates it, what is the format (email/letter etc)?

Are there any specific resources for Aboriginal and Torres Strait Islander students and their parents/caregivers?

Is there any additional support provided to Aboriginal and Torres Strait Islander students in general for HPV vaccination?

[*If relevant*] Is there any additional teaching about the HPV vaccination in class for students?

Is there any dedicated parent/caregiver information session about the HPV vaccination?

How do students and parents/caregivers react to the information?

Are there any major differences in information and support for Dose 1 (which is combined with dTpa) and Dose 2?

Consent

*If possible, researcher to have copies of information and consent forms provided by the relevant school to parents and students to refer to.*

What role do you play in the provision of consent for vaccination at [school]? *Explore: how is it sent out? Are there reminders? How is it collected? What happens if the consent form is not returned (follow up? By who?) what do you do with the completed consent forms?*

*If applicable – how do boarders get parent/caregiver consent?*

Are you involved in the disclosure of student and parent information to the School Immunisation Provider? Can you describe how that process works?

How do you get access to the list of students whose parents have consented? Are there any challenges in this?

What happens if a student turns up for vaccination but does not have a completed consent form?

What happens if fields are missing/not completed on the consent form?

Is any action taken if the Indigenous status field is left blank? Would the student be asked at the point of vaccination?

Can you tell me about any support provided to Aboriginal and Torres Strait Islander students and parents/caregivers to complete consent forms?

Do you feel that parents/caregivers have a choice in whether their child receives the HPV vaccine? *Why do you say that?*

What about the students – do you feel they have a choice in whether they receive the HPV vaccine? *Why do you say that? How do you feel about the idea of students having a choice?*

Program delivery at the school

Tell me about the logistics of the day of the vaccination. Can you walk me through vaccination day and your role in it? *Explore as timeline of events – where, when are they held, who is responsible, how do students arrive, how do students act/feel)*

Which council or HHS provides the vaccination?

Who/which organisation administers the vaccination?

How are adverse reactions managed and monitored?

Can you tell me about the school absenteeism rates on vaccination day?

What happens if a child with parent/caregiver consent misses out on Dose 1, 2 or both doses? *Prompt: are they followed up the previous year? Any challenges with this?*

Describe any follow-up about the HPV vaccine with students and parents/caregivers. *Prompts: are parents/caregivers informed if child was vaccinated/missed out or if they had an adverse reaction; any further education or information provided afterwards?*

Please describe any policies and procedures in place to support Aboriginal and Torres Strait Islander students and their parents/caregivers around HPV Vaccination?

Are there any major differences in how the program is delivered for Dose 1 (which is combined with dTpa) and Dose 2?

Pandemic

Was there anything different about how the HPV vaccination was delivered this year compared to last year due to the pandemic?

Describe any impacts the COVID-19 pandemic had on the delivery of HPV vaccination at this school.

Improving HPV vaccination

Does the HPV vaccination program meet the needs of students? Why do you say that?

Do you feel that Aboriginal and Torres Strait Islander students are supported in the school’s HPV vaccination program?

If you could change anything about how the HPV vaccination program is run at this school, is there anything you would do differently? *Explore.* Prompts: *Is there anything you would change that affects Aboriginal and Torres Strait Islander students? Why would you change these things?*

What would you do instead/what would work better?

Describe anything you think is working well in supporting students to have the HPV Vaccination? Prompts: *Is there any strategy that is supporting Aboriginal and Torres Strait Islander students? Why do you think these things are working well?*

To what extent would you say that equitable HPV vaccination is the responsibility of your role?

If it was your job to ensure over 90% vaccination coverage was achieved at this school, how would you go about this?

Conclusion

Is there anything else you want to say about HPV vaccinations – at this school or more generally?

**WRAPPING UP**

That’s it for our yarn today. I’m stopping the recording now.

PRESS STOP ON THE RECORDER NOW
