# Supplementary material for: Stakeholder perspectives on HPV vaccination uptake among Aboriginal and Torres Strait Islander adolescents via the school immunisation programmes in Queensland: a qualitative study
Source: BMJ Open. 2025 Jun 4;15(6):e097518. doi: 10.1136/bmjopen-2024-097518 (PMC12142122; doi:10.1136/bmjopen-2024-097518)
Supplement: Supplementary file 4 [file bmjopen-2024-097518.reviewer_comments.pdf]

PEER REVIEW HISTORY

BMJ Open publishes all reviews undertaken for accepted manuscripts. Reviewers are asked to complete a checklist review form and are provided with free text boxes to elaborate on their assessment. These free text comments are reproduced below.

ARTICLE DETAILS

Title (Provisional)

A qualitative study on Stakeholder perspectives on HPV vaccination uptake among Aboriginal and Torres Strait Islander adolescents via the School Immunisation Program in Queensland

Authors

Morseu-Diop, Ami; Butler, Tamara; Anderson, Kate; Brotherton, Julia; Cunningham, Joan; Jaure, Allison; Garvey, Gail; AhWing, Evan; Clements, Vanessa; Egert, Sonya; Lomas, Frances; Ross, Casey; Whop, Lisa J

VERSION 1 - REVIEW

**Reviewer**	**1**
**Name**	**Gwynne, Kylie**
**Affiliation**	**Macquarie University**
**Date**	**13-Jan-2025**
**COI**	**None**

Thank you for the opportunity to review this interesting and important qualitative research about school-based HPV vaccination for Aboriginal and Torres Strait Islander young people. The manuscript is well written. I offer the following minor suggestions:

1. Quotes from respondent #4156 in the scheduling and data sharing sections appear to be from the perspective of the immunisation team rather than the school. Can you please clarify?

2. There are some words missing in the first sentence of the discussion.

**Reviewer**	**2**
**Name**	**Mantina, Namoonga**
**Affiliation**	**The University of Arizona Mel and Enid Zuckerman College of Public Health**
**Date**	**01-Feb-2025**

**COI**	**None**

Thank you for submitting this important work. Great appreciation for the community participatory engagement incorporated throughout the research process.

line 23-24: the statement "there has been higher HPV vaccine initiation" its unclear what the comparison is for the values subsequently provided. is it pre 2020 vs post 2020? please clarify what the baseline is

did data analysis happen concurrently with data collection? Its mentioned on page 10 line 9 that data data collection continued after data saturation was achieved. how was data saturation determined?

page 20 line 12- there were comments left in the document

**Reviewer**	**3**
**Name**	**Reilly, Rachel**
**Affiliation**	**South Australian Health and Medical Research Institute, Wardliparingga**
**Date**	**23-Feb-2025**
**COI**	**None**

Thank you for the opportunity to review this paper. It addresses an important topic and is generally well written. Achieving equity in HPV vaccination rates is clearly very important, and the authors articulate the need for the study well.

Overarching concerns

Overall, while the paper follows a logical structure, I found the relationship between different sections a little disjointed – I think perhaps because it is only one component of the larger project outlined in the protocol, which also includes data from Aboriginal and Torres Strait Islander adolescents and their families/caregivers. It is more challenging for this data to stand on its own, and I think an explanation for the decision to present this component separately would help to contextualise the findings.

This is particularly true given the emphasis on Indigenist research methods, which are defined in the paper as 'research by Indigenous people whose primary informants are Indigenous people…' and described in the protocol as privileging Aboriginal and Torres Strait Islander voices. The use of these methods, alongside the majority Aboriginal and Torres Strait Islander research team and governance group, is a unique strength of the paper, however it is difficult to interpret how this approach was borne out in practice. The paper would be strengthened by a more detailed explanation of how these methods were applied in this specific component of the larger project, particularly for an international audience.

Similarly, the authors mention in the methods that the work was guided by an ecological model, but this is not mentioned again, and it is not clear how it guided the analysis. It would be helpful to outline in the methods and discussion how this model was applied.

With some revision, I think these issues can be addressed sufficiently for publication.

Specific comments

- To assist with interpretation of the findings and recommendations it would be helpful to include a description of the immunisation program itself, and the processes that are followed when state health employees visit schools to provide immunisations to students. This may not be immediately clear to an international audience, particularly given the geographic diversity of the schools included.

- The phrase 'maximally diverse' is used in the abstract to describe the ten schools recruited, and in the methods to describe the desired sample. It would be good to explain what characteristics this diversity is referring to.

- For clarity, I think it would be good to use consistent terms to describe 'key informants' or 'school representatives' (I think these are the same?)

- Some additional detail about the yarning method would be helpful in the data collection method, so that the methods may be considered replicable.

- The authors state that transcripts were not returned to participants for comment or correction – why is this? Was this offered but declined?

- The number of people who declined to participate was not recorded – why is this? If it was not practical/possible to do this, it would be good to say this.

- Check journal requirements reporting version of N Vivo.

- Re thematic analysis, was any guidance or theoretical approach adopted here? Great to provide a reference. Did the Indigenist research or ecological model influence the analysis?

- The protocol mentions 10 schools and 3-6 local key informant participants per school. The resultant number is much smaller than this. Why is this? Was data saturation reached earlier than anticipated? There were no teachers or principals in the sample – was this deliberate?

- The authors write that the 'coding summaries were reviewed…. and written for publication by AMD.' - I'm not sure if this is meant to refer to themes? Also, I'm not sure if the authors intended to imply that publication was the primary goal of the analysis?

- The results are described quite well, and quotes provide good illustrations of the themes in most cases. Some queries:

o Under 'clinic venue adherence health and safety requirements' the finding that 'providers valued being regarded as competent…' does not seem to fit.

o Under ‘supporting Aboriginal and Torres Strait Islander families through the vaccination pathway,’ the first point is about linking Aboriginal and Torres Strait Islander with supports, and the quote indicates that ‘the more Indigenous enrolments we have the harder it is with these things’ – referring to returning consent forms. It would be good to perhaps offer a little more context here to move away from an implicit deficit discourse.

o Under resources for Aboriginal and Torres Strait Islander families’ there is a section on issues with language and consent forms. I wonder if this could be linked with the larger earlier section on consent? Also, the emphasis here seems to be on locating the problem with parents and caregivers lack of knowledge. I think a slight re-orientation of the problem to the system/environment – eg information about HPV vaccination has not been available to parents/caregivers for many reasons – might fit better with the intended ecological orientation of the work and the suggested response.

o There is a number provided with each quote- is this meant to be meaningful to the reader? An explanation would be helpful.

The discussion outlines some sensible responses to the challenges identified by the participants.

- Although the authors state that HPV vaccination is everybody’s business, and schools are part of a larger community, the onus for many of the suggested changes does seem to be on what schools should do differently. I wonder if a consideration of broader ecological factors could be appropriate here – eg what are the broader environmental factors that may be driving inequities? What lies behind some of the logistical problems and resourcing shortfalls?

- Although the findings are presented as though these challenges extend across all schools, whether they are in metropolitan, regional or remote locations, it is difficult to imagine that the issues and challenges are the same given the very different contexts in which these schools operate. Some recognition of how the findings and recommendations may apply differently across contexts may be appropriate.

- Suggest updating the section on Covid-19 as the latest HPV data shows an increase in vaccination rates.

- In the strengths and limitations, it would seem appropriate to mention that the findings are limited by not including voices of Aboriginal and Torres Strait Islander young people and their caregivers. When available, this additional data will provide a much richer understanding of the issues surrounding HPV vaccination.

- It would be good to provide some more information about research translation. Is there a plan to implement the recommendations?

- There are some minor typographical errors – check for missing spaces, spelling (sterring), and comments left behind.

VERSION 1 - AUTHOR RESPONSE

	Reviewer comment	Response and relevant text excerpt	Page number
**2.1**	**Reviewer 1.**<br>Thank you for the opportunity to review this interesting and important qualitative research about school-based HPV vaccination for Aboriginal and Torres Strait Islander young people. The manuscript is well written. I offer the following minor suggestions:	Thank you for the positive comments.	-
**2.2**	Quotes from respondent #4156 in the scheduling and data sharing sections appear to be from the perspective of the immunisation team rather than the school. Can you please clarify?	Respondent #4156 was in an administrative role within the immunisation team. We have now rectified this within the manuscript.	Pg 11
**2.3**	There are some words missing in the first sentence of the discussion.	This has been corrected. It now reads:<br>This study aimed to understand the perspectives of immunisation program providers and school staff (key stakeholders) involved in, and responsible for, delivering the school-based immunisation program (SBIP) adolescents in Queensland, with a focus on HPV vaccination uptake among Aboriginal and Torres Strait Islander adolescents.	Pg 17
**3.1**	**Reviewer 2.**<br>Thank you for submitting this important work. Great appreciation for the community participatory engagement incorporated throughout the research process.	Thank you for the positive feedback.	-
**3.2**	line 23-24: the statement "there has been higher HPV vaccine initiation" its unclear what the comparison is for the values subsequently provided. is it pre 2020 vs post 2020? please clarify	This statistic reflects post 2020 rates. We have amended to be clearer (Background section, paragraph 2).<br>Vaccination initiation rates for Aboriginal and Torres Strait Islander adolescent males and females peaked in 2020 (83% v 87%), however,	Pg 4

	what the baseline is	since 2021 this has continued to decline.	
**3.3**	did data analysis happen concurrently with data collection? Its mentioned on page 10 line 9 that data collection continued after data saturation was achieved. how was data saturation determined?	We have clarified that data analysis commenced after data collection conclusion in the first sentence of the Data analysis section (pg 8):<br>Analysis commenced after data collection concluded.<br>We have clarified our approach to data saturation in this added text (last sentence of Recruitment and participants section, pg 7):<br>As we aimed to recruit a maximally diverse sample, data saturation was not a priority. However, when analysis commenced it was clear data saturation had been achieved.	Pg 7, 8
**3.4**	page 20 line 12- there were comments left in the document	We have removed any comments.	-
**4.1**	**Reviewer 3.**<br><br>Thank you for the opportunity to review this paper. It addresses an important topic and is generally well written. Achieving equity in HPV vaccination rates is clearly very important, and the authors articulate the need for the study well.	Thank you for the detailed comments and feedback, it is much appreciated.	-
**4.2**	Overall, while the paper follows a logical structure, I found the relationship between different sections a little disjointed – I think perhaps because it is only one component of the larger project outlined in the protocol, which also includes data from Aboriginal and Torres Strait Islander adolescents and their families/caregivers. It is more challenging for this data to stand on its own, and I think an explanation for the decision to present this component separately would help to contextualise the findings.	Where possible, we have provided further detail throughout the manuscript so that it is not solely referencing the original protocol. These stakeholders are an important component of understanding experiences and perspectives of those involved in promoting and delivering the HPV vaccination to Aboriginal and Torres Strait Islander adolescents.	-

**4.3**	This is particularly true given the emphasis on Indigenist research methods, which are defined in the paper as ‘research by Indigenous people whose primary informants are Indigenous people…’ and described in the protocol as privileging Aboriginal and Torres Strait Islander voices. The use of these methods, alongside the majority Aboriginal and Torres Strait Islander research team and governance group, is a unique strength of the paper, however it is difficult to interpret how this approach was borne out in practice. The paper would be strengthened by a more detailed explanation of how these methods were applied in this specific component of the larger project, particularly for an international audience.	We have added additional detail to address this comment under the Data Analysis section.<br>In line with Rigney’s Indigenist Research approach and as outlined in the Reflexivity section of this paper, it was important that the conceptualisation, data analysis, interpretation of findings, and writing were led by Aboriginal and Torres Strait Islander researchers. Under the governance of an Aboriginal and Torres Strait Islander steering committee, these principles were supported and upheld by all researchers on the team. Steering committee meetings were held several times a year throughout the duration of the project. Members provided valuable guidance on the interview guide, ideas for school reciprocity, and interpretation of results. Furthermore, members had the option to be recognised as co-authors. These principles were particularly important to apply in this sub-analysis from a predominantly non-Indigenous key stakeholder group, to appropriately unpack the power dynamics, and structural barriers discussed and to ensure that the focus was on delivering the vaccine to Aboriginal and Torres Strait Islander adolescents.	Pg 8
**4.4**	Similarly, the authors mention in the methods that the work was guided by an ecological model, but this is not mentioned again, and it is not clear how it guided the analysis. It would be helpful to outline in the methods and discussion how this model was applied. With some revision, I think these issues can be addressed sufficiently for publication.	We have clarified in the Data Analysis section that the ecological model of health promotion was used to deductively generate codes during thematic analysis:<br>Codes were generated inductively from the data and deductively using an ecological model of health promotion (18).<br>Section – Data Analysis (pg 8, paragraph 3)<br>The application of an ecological model of health for this analysis, allowed for an exploration of factors occurring at the individual level (Immunisation providers and school staff knowledge and attitudes), interpersonal level (relationships between families, schools, and immunisation providers), institutional level (school policies, program design, coordination between sectors), and broader system level contributors (historical and colonial legacies, public health policies).<br>We have also added the following to the Discussion section (pg 21, last paragraph):<br>The ecological model applied to our analysis illustrates the importance of cross-sectoral	Pg 8, 21

		strategies that address school-level operational issues but also structural inequities that contribute to lower HPV vaccine uptake and acceptance among Aboriginal and Torres Strait Islander adolescents and their caregivers.	
**4.5**	To assist with interpretation of the findings and recommendations it would be helpful to include a description of the immunisation program itself, and the processes that are followed when state health employees visit schools to provide immunisations to students. This may not be immediately clear to an international audience, particularly given the geographic diversity of the schools included.	We have clarified some of the text in the first paragraph of the Background section to provide some further context about the immunisation program.<br><br>HPV vaccination has been available since 2007 through Australia's National Immunisation Program (NIP), which can be accessed for free via school-based immunisation programs (SIP), that are coordinated by State and Territory health departments.<br><br>We have also added some text under the Background section (pg 5), giving a brief overview of some of the roles, and processes followed pre, during, and post vaccination day.<br><br>Some of the roles of immunisation providers involved in delivering the SIP include, coordinating and planning vaccination schedules with schools, educating and communicating with parents, students, and school staff about the vaccines and addressing concerns, and managing adverse events on vaccination day. Post-vaccination generally includes updating student records on the Australian Immunisation Register (AIR) and organising catch-up clinics for students who missed the scheduled vaccination dates. School staff involved in delivering the SIP share similar duties, particularly around providing information to parents and students, distributing and obtaining consent forms, managing students' emotions on vaccination day and following up with absent students.	Pg 4, 5
**4.6**	The phrase 'maximally diverse' is used in the abstract to describe the ten schools recruited, and in the methods to describe the desired sample. It would be good to explain what characteristics this diversity is referring to.	We have clarified the meaning of maximally diverse in the second paragraph under Recruitment and Participants (pg 7):<br><br>…. school recruitment aimed to achieve a maximally diverse sample across Queensland (including metropolitan, regional, remote districts, government, and independent schools, and varying school size).<br><br>We have included School size and school sector to Table 1 under Results to further highlight the	Pg 7, 9

		diversity of the schools from which participants were recruited (see pg 9).	
**4.7**	For clarity, I think it would be good to use consistent terms to describe ‘key informants’ or ‘school representatives’ (I think these are the same?)	We have added the following text in the recruitment and participants section, defining each group.<br><br>We use key informants to refer to both providers and school staff collectively.<br><br>We have clarified the distinct role the School Representative played in the recruitment process with the below text (second paragraph of Recruitment and participants section):<br><br>Potential participants were initially identified by the nominated School Representative at each participating school. The School Representative provided advice on local protocols and appropriate research approach, or this was sought from other Aboriginal and Torres Strait Islander members.	Pg 7
**4.8**	Some additional detail about the yarning method would be helpful in the data collection method, so that the methods may be considered replicable.	The larger project is titled “Yarning about HPV Vaccination” to highlight its focus on HPV vaccination of Aboriginal and Torres Strait Islander people. Yarning is an appropriate qualitative data collection method for Aboriginal and Torres Strait Islander peoples. However, given few of the participants in this sub-study were Aboriginal and/or Torres Strait Islander, we used semi-structured interviews to collect qualitative data. As such a description of Yarning methods is not necessary for this sub-study.<br><br>We have clarified this with the below text: (first paragraph of Data Collection).<br><br>While Yarning methods were used for other participant groups in the larger project, few key informants in this sub-study identified as Aboriginal and Torres Strait Islander people and so semi-structured interviews were used.	Pg 7
**4.9**	The authors state that transcripts were not returned to participants for comment or correction – why is this? Was this offered but declined?	We have clarified that transcripts were not returned to participants due to logistical reasons (see last paragraph of Data collection):<br><br>Transcripts were not returned to participants for comment or correction as it was not logistically feasible. No participant dropped out or withdrew statements from the study throughout the duration or completion of the project.	Pg 8

**4.10**	The number of people who declined to participate was not recorded – why is this? If it was not practical/possible to do this, it would be good to say this.	We have clarified this issue with the below additional text:<br>Section – Recruitment and participants (pg 7, paragraph 2)<br>The School Representative then passed on their contact details to the research team if they expressed interest in participating. Because participants chose to take part by contacting the research team, we were unable to record how people declined to participate.<br>We were unable to record how many people declined to participate due to practical reasons.<br>We have moved this information to the Participants and Recruitment section as the reason for not recording the number who declined was linked to the recruitment method.<br>As mentioned in response to comment 4.9, we have clarified the text regarding participant withdrawal within the Data Collection section (pg 8, last paragraph).<br>No participant dropped out or withdrew statements from the study throughout the duration or completion of the project.	Pg 7, 8
**4.11**	Check journal requirements reporting version of N Vivo.	We could not find specific journal requirements regarding this matter. We have included the version number (Data Analysis section, line 2):<br>Data from interviews and field notes were imported into the software NVivo (Version 13) to facilitate analysis.	Pg 8
**4.12**	Re thematic analysis, was any guidance or theoretical approach adopted here? Great to provide a reference. Did the Indigenist research or ecological model influence the analysis?	We've clarified this under the Data analysis section (paragraph 2).<br>In line with Rigney's Indigenist Research approach and as outlined in the Reflexivity section of this paper, it was important that the conceptualisation, data analysis, and interpretation of findings, were led by Aboriginal and Torres Strait Islander researchers, guided by an Aboriginal and Torres Strait Islander steering committee, and these principles supported and upheld by non-Indigenous researchers on the team. These principles were particularly important to apply in this sub-analysis from a predominantly non-Indigenous key informant group, to appropriately unpack power dynamics, and structural barriers discussed and to ensure	Pg 8

		that the focus of delivering the vaccine to Aboriginal and Torres Strait Islander students.<br><br>The application of an ecological model of health for this analysis, allowed for an exploration of factors occurring at the individual level (Immunisation providers and school staff knowledge and attitudes), interpersonal level (relationships between families, schools, and immunisation providers), institutional level (school policies, program design, coordination between sectors), and broader system level contributors (historical and colonial legacies, public health policies).	
**4.13**	The protocol mentions 10 schools and 3-6 local key informant participants per school. The resultant number is much smaller than this. Why is this? Was data saturation reached earlier than anticipated? There were no teachers or principals in the sample – was this deliberate?	The reason for the low number of key informants per school is due to the timing of the data collection which was partly conducted during the COVID-19 pandemic and disruptions resulting in school lock downs and limited availability of immunisation nurses and coordinators. We have noted this in the Strengths and Limitations section (paragraph 3).<br><br>As noted in the Recruitment and Participants section, Principals and Teachers were invited to participate in the study. We are unable to state how many due to the small sample size being potentially identifying. Therefore, they are represented by the category “school staff”.<br><br>As qualitative data strives for richness and depth of data rather than representativeness achieved through large sample sizes, the sample size adequately captures a wide range of relevant views.	Pg 22
**4.14**	The authors write that the ‘coding summaries were reviewed…. and written for publication by AMD.’ - I’m not sure if this is meant to refer to themes? Also, I’m not sure if the authors intended to imply that publication was the primary goal of the analysis?	That intended reason for the statement was to highlight that the codebook was reviewed by Aboriginal and Torres Strait Islander researchers before it was finalised for use in interpreting the data and themes. We have amended this to be clearer.<br><br>Coding summaries were reviewed by TB, LJW, and KA before use in interpreting the data and themes, with the final version of the codebook written by AMD.	Pg 7
**4.15**	The results are described quite well, and quotes provide good illustrations of the themes in most cases. Some queries:	This refers to the lack of trust and knowledge surrounding program providers role in administering and coordinating the vaccination on the day, to the extent that adherence to clinical requirements was neglected. We have reworded	Pg 11

	Under 'clinic venue adherence health and safety requirements' the finding that 'providers valued being regarded as competent…' does not seem to fit.	this section to be clearer.<br><br>Section – Clinic venue adherence health and safety requirements<br><br>Providers valued being regarded by schools as competent in their role and understanding the safety requirements of their duties during clinic visits.	
**4.16**	Under 'supporting Aboriginal and Torres Strait Islander families through the vaccination pathway,' the first point is about linking Aboriginal and Torres Strait Islander with supports, and the quote indicates that 'the more Indigenous enrolments we have the harder it is with these things' – referring to returning consent forms. It would be good to perhaps offer a little more context here to move away from an implicit deficit discourse.	Thank you for this comment. As this participant states afterwards that: "having consistency and support absolutely that work in community and with families and with the school is a vital link" [1563, Immunisation Coordinator, Remote], we believe that the focus on identifying factors that support returning of consent forms minimises the risk of implied deficit discourse. To improve clarity and provide more context we have included the following text with a quote.<br><br>Section – Supporting Aboriginal and Torres Strait Islander families through the vaccination pathway<br><br>Having enough lead time to engage with families was regarded as being beneficial.<br><br>*'…when we're working with Aboriginal and Torres Strait Islander communities, we talk about the three cups of tea; the first time is just the meet and greet, the second time is that you're now all of a sudden friends, and then the third time is you're family, we'll do anything for you. It's the same situation [with following-up on consent forms]. It needs a longer lead-in time' [1662, Student Support Staff].*<br><br>One participant reflected that this can be challenging when there is a large cohort of Aboriginal and Torres Strait Islander students, and adequate time and resources are not embedded in the process.	Pg 13
**4.17**	Under resources for Aboriginal and Torres Strait Islander families' there is a section on issues with language and consent forms. I wonder if this could be linked with the larger earlier section on consent? Also, the emphasis here seems to be on locating the	Whilst we agree with you, this is beyond the scope of the paper.<br><br>The section referring to consent forms in the "Health literacy and education among caregivers" section is discussing the content of the consent forms and ensuring this is understandable for Aboriginal and Torres Strait Islander families. In contrast, the earlier section about consent forms	Pg 16

	problem with parents and caregivers lack of knowledge. I think a slight re-orientation of the problem to the system/environment – eg information about HPV vaccination has not been available to parents/caregivers for many reasons – might fit better with the intended ecological orientation of the work and the suggested response.	("Completing and returning consent forms" focuses on the logistical and practical factors surrounding physically returning the consent forms. Although related, we view these sections as addressing different aspects of the issue.<br>We have included the text below to highlight the role of the broader systemic barriers.<br>Section - HPV vaccination resources for Aboriginal and Torres Strait Islander families<br><u>Participants discussed how the lack of access to culturally appropriate information and resources, coupled with complex consent processes influenced informed decision making among Indigenous caregivers. Language barriers and diverse literacy levels meant that some Aboriginal and Torres Strait Islander caregivers had a limited understanding of the consent form and were not always sure what they were signing or consenting to.</u>	
**4.18**	There is a number provided with each quote- is this meant to be meaningful to the reader? An explanation would be helpful	We have used numbers to distinguish that there are different providers and school representatives speaking. We have decided to keep the numbers but have included the following text:<br>Section – Results (paragraph 2)<br><u>Four-digit participant identification codes are provided with quotes.</u>	Pg 10
**4.19**	The discussion outlines some sensible responses to the challenges identified by the participants.<br>- Although the authors state that HPV vaccination is everybody's business, and schools are part of a larger community, the onus for many of the suggested changes does seem to be on what schools should do differently. I wonder if a consideration of broader ecological factors could be appropriate here – eg what are the broader environmental factors that may be driving inequities? What lies behind some of the logistical problems and resourcing shortfalls?	We agree that broader ecological factors do play a role in shaping HPV vaccine coverage. The emphasis on school-level challenges highlights where participants (including school representatives) felt the most immediate change could occur. However, we recognise it is important to situate these challenges within the broader social and institutional context. We have included the text below to be clearer.<br>Section - Engaging and supporting Aboriginal and Torres Strait Islander adolescents and caregivers (paragraph 2)<br><u>Similarly, government accountability and greater investment is critical to ensure that schools are adequately resourced, and that all school staff are provided appropriate training – in terms of cultural safety and the logistical delivery of the SIP. There is also a need to recognise how schools have not historically been considered a safe or</u>	Pg 20

		inclusive environment by many Aboriginal and Torres Strait Islander peoples (28) and this continues to influence how Indigenous families engage with the education system.	
**4.20**	Although the findings are presented as though these challenges extend across all schools, whether they are in metropolitan, regional or remote locations, it is difficult to imagine that the issues and challenges are the same given the very different contexts in which these schools operate. Some recognition of how the findings and recommendations may apply differently across contexts may be appropriate.	Under the Strengths and Limitations section (line 8) we state that, "Additional research in other states is required to capture the perspectives and experiences of school staff and providers, and how uptake is impacted in other cohorts of Aboriginal and Torres Strait Islander adolescents and their caregivers."<br><br>The findings are not intended to be representative of extending across all schools, however, it aligns with much of the existing literature on school-based immunisation programs. We have amended this to be clearer.<br><br>Section – Recommendations<br><br>As previously mentioned, further research is recommended to explore state specific differences in the school-based HPV vaccination program.<br><br>Section – Strengths/Limitations (paragraph 2)<br><br>We recognise that there are diverse school contexts and so the challenges presented, and potential solutions will vary. Governance structures and funding allocation also differ between states and territories, so recommendations must be tailored to the specific needs and capacities of different communities.	Pg 22
**4.21**	Suggest updating the section on Covid-19 as the latest HPV data shows an increase in vaccination rates.	This is incorrect. The latest data shows that there is a decrease in HPV coverage rates. We have included extra text to be clearer.<br><br>Section – COVID-19 pandemic and learnings for future disruptions<br><br>In 2022, 83% and 78.1% of Indigenous boys and girls, respectively, received one dose of HPV vaccine by age 15, decreasing in 2023 for both groups (80.9% v 75%), with even lower coverage observed by jurisdiction of residence.<br><br>Accelerated catch-up efforts and strategies to minimise both long and short-term impacts of SIP disruptions is crucial, as delays would result in further preventable cervical cancer cases in future (35).	Pg 21

**4.22**	In the strengths and limitations, it would seem appropriate to mention that the findings are limited by not including voices of Aboriginal and Torres Strait Islander young people and their caregivers. When available, this additional data will provide a much richer understanding of the issues surrounding HPV vaccination.	We have included that a limitation of the study was that most of the Key Stakeholders did not identify as Aboriginal and/or Torres Strait Islander. As we have noted, another publication is currently under review that focuses on the views of Aboriginal and Torres Strait Islander parents and adolescents. To clarify the focus of the current study, we have included the text below at the end of the Background section.<br><br>The aim of this study is to explore the perspectives and experiences that program providers and school staff involved in the delivery of school-based HPV vaccination programs for Aboriginal and Torres Strait Islander adolescents in Queensland. It is one component of a larger study (13), which recruited 65 Aboriginal and Torres Strait Islander adolescents and 20 caregivers across 10 schools, to understand their experiences engaging with the HPV vaccination program. We encourage readers to engage with this paper in conjunction with the broader study, for a comprehensive view.	Pg 6
**4.23**	It would be good to provide some more information about research translation. Is there a plan to implement the recommendations?	We have provided a list of recommendations in the manuscript, that will be disseminated as part of our research translation to relevant key stakeholders.	
**4.24**	There are some minor typographical errors – check for missing spaces, spelling (steering), and comments left behind.	All typos and errors have been corrected and all comments removed.	

VERSION 2 - REVIEW

Reviewer 3

Name Reilly, Rachel

Affiliation South Australian Health and Medical Research Institute, Wardliparingga

Date 06-May-2025

COI

I commend the authors for their thorough and considered responses to each of the reviewers' comments.

I have only one further minor revision - the statement: "In 2022, 83% and 78.1% of Indigenous boys and girls, respectively, received one dose of HPV vaccine by age 15, decreasing in 2023 for both groups (80.9% v 75%), with even lower coverage observed by jurisdiction of residence." requires a reference, noting it appears to differ from the data published by AIHW, which was the basis of my initial comment: https://www.health.gov.au/topics/immunisation/immunisation-data/human-papillomavirus-hpv-immunisation-data.

VERSION 2 - AUTHOR RESPONSE

	**Reviewer comment**	**Response and relevant text excerpt**	**Page number**
**1.1**	**Reviewer 3.**<br>I commend the authors for their thorough and considered responses to each of the reviewers' comments. I have only one further minor revision - the statement: "In 2022, 83% and 78.1% of Indigenous boys and girls, respectively, received one dose of HPV vaccine by age 15, decreasing in 2023 for both groups (80.9% v 75%), with even lower coverage observed by jurisdiction of residence." requires a reference, noting it appears to differ from the data published by AIHW, which was the basis of my initial comment: https://www.health.gov.au/topics/immunisation/immunisation-data/human-papillomavirus-hpv-immunisation-data.	Thank you for the feedback. We are pleased that our responses to the initial review were satisfactory and appreciate all of the reviewer's feedback.<br>We have now included a reference for this statement to the NCIRS Annual Immunisation Coverage Report, which was also cited in the Introduction section of the paper.<br>We are aware of the discrepancy between NCIRS and AIHW data and understand it has been raised with the relevant departments but not yet addressed. The discrepancy in numbers is because the data on the Australian Government DoHAC website applies a two-dose definition of coverage up until 2022, rather than comparing single dose coverage (now accepted as providing sufficient protection) over time. Additionally, the methods used for the Australian Government DoHAC website estimates do not align with the standard reporting methods used by NCIRS. For these reasons we prefer to report NCIRS data.	21, line 9
		We have also replaced reference no. 31, cited under Culturally sensitive resources on page 21, to reflect the recently published HPV resources audit which includes co-authors of this manuscript and more accurately reflects the need for culturally sensitive resources. This has been updated in the reference list on pg 26.<br>31. Butler, T.L., et al., *An evaluation of Human Papillomavirus vaccination resources available*	Pg 26

		to Aboriginal and Torres Strait Islander adolescents and parents and caregivers in Australia. Patient Education and Counseling, 2025: p. 108820.	
